# Supplementary material for: Gendered economic, social and health effects of the COVID-19 pandemic and mitigation policies in Kenya: evidence from a prospective cohort survey in Nairobi informal settlements
Source: BMJ Open. 2021 Mar 3;11(3):e042749. doi: 10.1136/bmjopen-2020-042749 (PMC7931215; doi:10.1136/bmjopen-2020-042749)
Supplement: Author's manuscript [file bmjopen-2020-042749.draft_revisions.pdf]

BMJ Open is committed to open peer review. As part of this commitment we make the peer review history of every article we publish publicly available.

When an article is published we post the peer reviewers' comments and the authors' responses online. We also post the versions of the paper that were used during peer review. These are the versions that the peer review comments apply to.

The versions of the paper that follow are the versions that were submitted during the peer review process. They are not the versions of record or the final published versions. They should not be cited or distributed as the published version of this manuscript.

BMJ Open is an open access journal and the full, final, typeset and author-corrected version of record of the manuscript is available on our site with no access controls, subscription charges or pay-per-view fees (http://bmjopen.bmj.com).

If you have any questions on BMJ Open's open peer review process please email info.bmjopen@bmj.com

Gendered social, health and economic effects of the COVID-19 pandemic and mitigation policies in Kenya: evidence from a prospective cohort survey in Nairobi informal settlements

Journal:	*BMJ Open*
Manuscript ID	bmjopen-2020-042749
Article Type:	Original research
Date Submitted by the Author:	14-Jul-2020
Complete List of Authors:	Pinchoff, Jessie; Population Council, Poverty, Gender and Youth<br>Austrian, Karen; Population Council, Poverty, Gender & Youth<br>Rajshekhar, Nandita; Independent consultant<br>Abuya, Timothy; Populat Council Gen Accid Insurance House, Re; Population Council Kenya,<br>Kangwana, Beth; Population Council, Poverty, Gender & Youth<br>Ochako, Rhoune; Population Council, Poverty, Gender & Youth<br>Tidwell, James; World Vision International,<br>Mwanga, Daniel; Population Council, Poverty, Gender & Youth<br>Muluve, Eva; Population Council, Poverty, Gender & Youth<br>Mbushi, Faith; Population Council, Poverty, Gender & Youth<br>Nzioki, Mercy; Population Council, Poverty, Gender & Youth<br>Ngo, Thoai; Population Council, Poverty, Gender and Youth
Keywords:	Epidemiology < TROPICAL MEDICINE, Public health < INFECTIOUS DISEASES, PUBLIC HEALTH

SCHOLARONE™
Manuscripts

I, the Submitting Author has the right to grant and does grant on behalf of all authors of the Work (as defined in the below author licence), an exclusive licence and/or a non-exclusive licence for contributions from authors who are: i) UK Crown employees; ii) where BMJ has agreed a CC-BY licence shall apply, and/or iii) in accordance with the terms applicable for US Federal Government officers or employees acting as part of their official duties; on a worldwide, perpetual, irrevocable, royalty-free basis to BMJ Publishing Group Ltd ("BMJ") its licensees and where the relevant Journal is co-owned by BMJ to the co-owners of the Journal, to publish the Work in this journal and any other BMJ products and to exploit all rights, as set out in our licence.

The Submitting Author accepts and understands that any supply made under these terms is made by BMJ to the Submitting Author unless you are acting as an employee on behalf of your employer or a postgraduate student of an affiliated institution which is paying any applicable article publishing charge ("APC") for Open Access articles. Where the Submitting Author wishes to make the Work available on an Open Access basis (and intends to pay the relevant APC), the terms of reuse of such Open Access shall be governed by a Creative Commons licence – details of these licences and which Creative Commons licence will apply to this Work are set out in our licence referred to above.

Other than as permitted in any relevant BMJ Author's Self Archiving Policies, I confirm this Work has not been accepted for publication elsewhere, is not being considered for publication elsewhere and does not duplicate material already published. I confirm all authors consent to publication of this Work and authorise the granting of this licence.

For peer review only

Potential Title: Gendered social, health and economic effects of the COVID-19 pandemic and mitigation policies in Kenya: evidence from a prospective cohort survey in Nairobi informal settlements

Authors: Jessie Pinchoff PhD[1], Karen Austrian PhD[2], Nandita Rajshekhar MPH[3], Timothy Abuya PhD[2], Beth Kangwana PhD[2], Rhoune Ochako PhD[2], James B Tidwell PhD[4,5], Daniel Mwanga[2], Eva Muluve[2], Faith Mbushi[2], Mercy Nzioki[2], Thoai D Ngo PhD[1]

Author Information:

[1] Population Council, Department of Poverty, Gender and Youth, New York NY 10017
[2] Population Council – Kenya, Nairobi Kenya
[3] Independent consultant
[4] Harvard Kennedy School of Government, Cambridge, MA
[5] World Vision, Washington, DC

Corresponding Author:
Jessie Pinchoff, PhD
Population Council
Department of Poverty, Gender and Youth
One Dag Hammarskjold Plaza #3
New York, NY 10017
Email: jpinchoff@popcouncil.org

Word Count: 4,234

For peer review only

For peer review only - http://bmjopen.bmj.com/site/about/guidelines.xhtml

ABSTRACT

Objectives: COVID-19 may spread rapidly in densely populated urban informal settlements. Kenya swiftly implemented mitigation policies; we assess the social, health, and economic harm experienced by vulnerable households during the response, by gender.

Design: A prospective longitudinal cohort mobile-phone based study with repeated surveys in April, May and June.

Participants and Setting: 2,009 households across five informal settlements in Nairobi, sampled from participants of two previously interviewed cohorts.

Primary and Secondary Outcome Measures: Social, health, and economic effects of the pandemic, specifically food insecurity, risk of household violence, and forgoing necessary health services in the last three months were the three outcomes of interest. Linear probability regression models were constructed to determine factors associated with these outcomes, for the full sample and stratified by gender.

Results: In May, 43% of participants had lost full employment, and 74% reported skipping a meal. Women were 6 percentage points (pp) more likely to skip a meal vs men (coeff: 0·055; 95% Confidence Interval (CI) 0·016, 0·094), and those who had completely lost their income were 15 pp more likely vs those employed (coeff: 0·154; 95% CI 0·125, 0·184) to skip a meal. Compared to men, women were 8 pp more likely to report increased risk of household violence (coeff: 0·079; 95% CI 0·028, 0·130), and 6 pp more likely to forgo necessary healthcare (coeff: 0·057; 95% CI 0·038, 0·076). While those receiving assistance increased (7% in April to 21% in May), those reporting this assistance met their basic needs decreased (47% to 38%).

Conclusions: Key social and economic indicators are worse in May compared to April, and women bear a disproportionate burden. As mitigation measures continue, policymakers need to immediately deploy assistance targeted to the most vulnerable households in urban slums, with attention to women.

Key Words: COVID-19, gender, Kenya, urban, health, epidemiology

ARTICLE SUMMARY

Strengths and Limitations of this Study

- This is one of the first longitudinal cohort studies on COVID-19 vulnerability and risks in informal settlements in a sub-Saharan African city.
- These findings highlight the disproportionate risks and impacts shouldered by women in the COVID-19 pandemic, and identify key characteristics associated with food insecurity, risk of household violence, and forgoing necessary medical care by gender. These results will inform social support programs in response to this pandemic to ensure the most vulnerable receive targeted assistance, with attention to the needs of women.
- There are two limitations: first, since participants of this study were sampled from existing cohorts from ongoing adolescent studies, the study population may not be fully representative of the setting population. Households were only eligible if an adolescent resided in the home; therefore, some households are not represented. Second, the survey was conducted among those we were able to reach by mobile phone, and responses are self-reported, which can lead to reporting and selection bias.

For peer review only - http://bmjopen.bmj.com/site/about/guidelines.xhtml

INTRODUCTION

COVID-19, a highly transmissible respiratory disease caused by the novel severe acute respiratory syndrome coronavirus 2 (SARS-CoV2), was officially declared a pandemic on March 11th 2020 by the World Health Organization (WHO).[1] African governments and global health experts are projecting the potential for up to 190,000 COVID-19 deaths in Sub-Saharan Africa if containment and mitigation efforts fail.[2] Urban informal settlements in Africa are facing increased risk for rapid spread of coronavirus due to high population density, inadequate housing and sanitation facilities, and intense levels of social mixing.[3] Sub-Saharan Africa is rapidly urbanizing; 47% of urban residents in the region live in informal settlements that are ill equipped to handle a disease outbreak.[4,5] Coupled with the higher risks of disease transmission, poor informal settlement residents face the concurrent shock of devastating impacts of physical distancing policies and lockdowns, including closing of businesses, schools, and large gatherings. Mitigation policies may slow the transmission of the virus, but they come with a heavy social and economic toll on poor urban populations, particularly for women.[6] To date, there has been extensive theorizing regarding how COVID-19 will impact African populations, but little systematic research. This study is one of the first prospective cohort studies conducted during COVID-19 to understand the experiences of those residing in urban informal settlements, including a broad set of indicators and measures to holistically understand the interconnected short- and long-term effects of the pandemic.

In Kenya, the first case of COVID-19 was detected on March 13th and resulted in the immediate closure of schools and restaurants/bars and the prohibition of large gatherings. Two weeks later, on March 26th Kenya banned international flights.[7] On April 6th, the Nairobi Metropolitan Area and three counties in coastal Kenya were contained, restricting movement into and out of these counties. Many businesses and stores have closed as a result. Three months into the crisis, Kenya has confirmed 6,673 cases and 149 deaths related to COVID-19 (as of July 1st, 2020).[8] An estimated 60-70% of Nairobi's more than 4 million residents reside in urban slums.[9] From March 30th-31st 2020, Population Council's COVID-19 mobile phone-based survey among 2,009 informant settlement dwellers found 61% of respondents reported physical distancing measures would be challenging to follow, as it would risk their income.[10] The survey also found about one in five were worried about food shortages (22%) and about one in three were worried about job or income loss (34%).[10] Lack of income may be a significant challenge if prices for food and other critical needs go up, as news outlets are reporting. Recent reports express concerns that COVID-19 and Kenya's mitigation policies may lead to severe setbacks in nutrition, immunization, other diseases, and gender equity.[11,12]

Urban poor households are highly vulnerable during crises, and when faced with severe external shocks, are less able to cope with the health and financial impacts, as seen in previous case studies.[13] Implementing physical distancing and personal hygiene measures recommended by the World Health Organization[14] to curb the spread of the outbreak will be challenging if not impossible in urban slums.[15] While there is fear regarding the spread of COVID-19, the potential harm caused by income loss, food insecurity, and foregoing health services may be just as severe, particularly for women.[16] Experts have yet to determine the severity of economic and social impacts likely to result from disease prevention and containment methods. A holistic approach is needed to understand these interrelated secondary effects.

Historically, women have been at a disproportionately high-risk during health and economic crises. During the Ebola epidemic, researchers found that women's roles as health workers and household primary care-givers made them more vulnerable to severe disease, and noted that much of women's labor in health crises goes unpaid, adding to the economic burden that may result from COVID-19.[16] During Ebola in Sierra Leone, researchers found that women disproportionately lost their jobs compared to men and the related economic disruptions impacted girls' education with more girls being pressured to drop out over boys due to economic constraints.[17] Women also tend to take on more unpaid household labor such as cooking, cleaning, and childcare during times of crises. With physical distancing, there are concerns that gender-based violence (GBV) may increase. Distancing may lead to social isolation,

For peer review only - http://bmjopen.bmj.com/site/about/guidelines.xhtml

exacerbate mental health issues, and reduce the safety of victims. Stress and coping mechanisms such as increased alcohol consumption may also lead to more instances of violence.[18] In China, reports of domestic violence tripled during lockdown,[19] while in South Africa, reports have increased with 87,000 reports of domestic violence were recorded in the first week of lockdown alone.[20] These trends threaten the progress made toward gender equality and gender-based violence reduction efforts.

Access to healthcare has always been challenging for poor urban populations, and the pandemic may exacerbate these difficulties. People may not be able to afford healthcare due to unemployment from the pandemic, facilities may restrict patient volume to minimize infection risk, and even if healthcare is available, people may avoid seeking care due to fear of infection at clinics. There is also concern that quarantine is being perceived as a punitive measure. During Ebola, fear and low trust in the health system resulted in negative impacts on downstream health outcomes, particularly in women and children.[21,22] A recent report suggests 30 million children's lives may be at risk if secondary effects on health systems are similar for COVID-19 as they were for Ebola.[23] For example, shortly after Ebola there was a rise in measles cases, due to the drop in vaccinations caused by the crisis.[24] This is already being reported in Kenya; compared with 2019, under-5 OPD attendance and vaccination rates are significantly down.[25] Diverting resources to emerging threats can lead to neglecting other infectious diseases resulting in new waves of disease outbreaks and many lives lost. These lessons need to be considered when allocating resources for COVID-19 and instating containment policies.

The primary objective of this study is to assess the short-term social, health, and economic effects of COVID-19 and related mitigation measures among a prospective, longitudinal cohort of households sampled from five of Nairobi's informal settlements. This is one of the first cohort studies during COVID-19, developed with the aim of understanding the experiences of households and the dynamic effects of the pandemic as the situation evolves. We evaluated disparities by gender and how these are associated with the social, health and economic effects of the pandemic, as research from previous health and humanitarian crises suggest women are likely to be disproportionately impacted. Our findings will help develop and better target both short- and long-term policy and interventions.

METHODS

The Population Council, in collaboration with the Kenyan Ministry of Health, has conducted mobile phone surveys across five urban slums in Nairobi (Kibera, Mathare, Kariobangi, Dandora, and Huruma). The study methods have been described elsewhere.[10] In brief, the study participants were randomly sampled from two existing longitudinal cohort studies, the Adolescent Girls Initiative-Kenya (AGI-K) and NISITU. Prior to COVID-19, the AGI-K urban cohort in Kibera and Huruma repeatedly sampled 2,565 randomly selected households that had at least one adolescent resident. This cohort was part of a four-arm randomized controlled trial testing the impact of adolescent girls programs. This included a baseline data collection in 2015, a second round of data collection in 2017, and a third round in 2019.[26] The NISITU cohort consisted of 4,519 households randomly sampled from households in Kariobangi, Dandora, and Mathare informal settlements. NISITU is a quasi-experimental study evaluating the effects of a gender transformative program for girls, boys, and young men. The NISITU baseline was conducted in early 2018 and the second wave of data collection in late 2019. For both cohorts, an initial household listing was conducted in the study sites to create a sampling frame of households with an eligible adolescent for the study. The last round of data collection for each was recently completed (September 2019 for AGI-K and in January 2020 for NISITU), therefore phone numbers for each head of household were up to date. All households were eligible for inclusion as long as an adult was reached on the phone. We randomly sampled from all 7,500 households, stratified by informal settlement. The COVID-19 cohort consists of about 2,009 adult household members interviewed on March 30th-31st. We re-interviewed 1,768 of these on April 13-14th, and 1,750 on May 10-11th 2020. Due to ongoing physical distancing policies in Kenya, all surveys were conducted on the phone to protect both participants and surveyors from potential COVID-19 spread. Each survey lasted about 30 minutes, with some questions asked across all three surveys and some unique questions in each of the three surveys.

For peer review only - http://bmjopen.bmj.com/site/about/guidelines.xhtml

Survey Instruments

The questionnaires used adapted standardized questions wherever possible, such as questions from the Demographic and Health Survey and the WHO/UNICEF Joint Monitoring Program on water and sanitation. A total of 77 Kenyan surveyors were trained on conducting mobile phone surveys, and before each survey round a training session was held to review the new questions. The first round questionnaire included questions on basic demographics, awareness of COVID-19, knowledge, perceived risk, preventive behaviors being implemented, channels of information, and trustworthiness of each source. In round two, questions regarding social and economic effects on households were added, for example, loss of employment, skipping meals, household costs, and gender-based violence or tensions experienced in the household. In round three, more detailed questions were added; such as how often participants are skipping meals. Each surveyor completed 10-15 surveys per day, and all phone numbers were tried up to three times if not reached on the first attempt. Questions were framed to reduce bias as much as possible. Data was collected using Open Data Kit (ODK) and exported to Stata v15 for analysis.

Ethical Approval

We received expedited ethical approval via amendments to existing protocols to re-contact respondents for the first two rapid surveys given the urgent need for information during this pandemic. The ongoing COVID-19 KAP survey cohort was approved by the Population Council IRB (p936) and AMREF ESRC (P803/2020). A research permit was issued by the National Commission for Science, Technology and Innovation (NACOSTI) in Kenya (P/20/5010).

Participant Involvement

It was not possible to involve participants in the study design or interpretation of results, due to the rapid response required around COVID-19, the inability to engage face to face or hold events, and that no funds were allocated for this.

Questionnaires and reports are publicly available, with the full de-identified dataset available upon request.

Data Analysis

Data from AGI-K and NISITU 2019 surveys were merged with rounds one, two, and three of the COVID-19 survey for additional information on households characteristics. Key health, social, and economic variables were tabulated by survey round and gender. Key health variables focused on whether participants reported that in the last two weeks, they had required a health service (e.g., for malaria, acute illness, immunization, or others) but did not seek healthcare for themselves or their children. Key social variables included seeing family and friends less due to COVID-19, or taking on more chores such as cooking, cleaning, and childcare. Participants were asked if they experienced more arguing, tension, household violence, or fear their partner would harm them due to COVID-19 (four binary questions). For analysis, participants who said yes to one or more of these questions were considered at risk of household violence. Key economic variables included reporting increased food prices, loss of income/employment and skipping a meal due to the pandemic. Very few questions had missing responses.

Linear probability regression models were constructed to explore: 1) factors associated with skipping meals due to COVID-19, 2) factors associated with risk of household violence and 3) factors associated with forgoing any health services due to COVID-19. Bivariate models were constructed first followed by a fully adjusted model. Fully adjusted models control for gender, age, wealth quintile, and informal settlement where the participant resides. In the models for skipping meals and forgoing health services, data were available for both April and May; in these models, clustering by participant was specified to allow the errors for the same participant to be correlated across each round over time. For the household violence risk model, these data were only collected from one time point (May). Models were then run stratified by gender.

For peer review only - http://bmjopen.bmj.com/site/about/guidelines.xhtml

RESULTS

Characteristics of respondents

A total of 2,009 individuals were surveyed in March, 1,761 in April (88% of round one), and 1,745 in May (99% of round two participants; 87% of round one participants). Overall, participants were 63% female, with an average age of 36.3 (SD=11·4) (Table 1).

Table 1: Demographic characteristics of study sample selected in March 2020

**Demographics**	**N (%)**
Total sample size round 1	2,009
Gender	
Female	1258 (62·8)
Male	747 (37·2)
Age	
Mean (SD)	36·3 (11·4)
Slum	
Dandora	459 (22·9)
Huruma	275 (13·7)
Kariobangi	414 (20·6)
Kibera	440 (21·9)
Mathare	416 (20·7)
Education	
No School	73 (3·6)
Primary	793 (39·6)
Secondary	878 (43·8)
Higher Education	257 (12·8)
Marital Status	
Married	1170 (58·5)
Single	502 (25·1)
Divorced/Separated Widowed	328 (16·4)

For peer review only

Most had completed secondary school (44%) and most were married (59%). Between April and May 2020, significant changes in some social, health, and economic effects were noted. Almost half of participants reported complete loss of income (43% in April vs 36% in May), experiencing more violence in the household (5% in April vs 3% in May), and increased reporting of skipping meals in the last week due to COVID-19 (74% vs 68%) (Table 2).

For peer review only - http://bmjopen.bmj.com/site/about/guidelines.xhtml

Table 2: Social, health and economic effects of mitigation measures in April (Round 2) and May (Round 3)

	April (Round 2)	May (Round 3)	p-value
Social effects			
See family less	992 (56·2)	986 (56·3)	0·946
See my friends less	1538 (87·1)	1588 (90·7)	0·001
Risk of household violence			
More tensions in the household	647 (36·6)	601 (34·3)	0·166
More violence experienced inside the house	58 (3·3)	86 (4·9)	0·019
More time arguing in the household	··	450 (25·7)	NA
More fear partner will harm you	··	103 (5·9)	NA
Combined into risk of household violence	··	743 (42·6%)	NA
Household chores			
More housework (general)	1081 (61·2)	··	NA
More time spent cooking	··	693 (39·6)	NA
More time spent cleaning	··	833 (47·6)	NA
More time spent taking care of children	··	960 (54·9)	NA
Neighborhood crime, violence			
Increased crime in the neighborhood	651 (36·9)	854 (48·8)	<0·001
More violence experienced outside the house	261 (14·8)	412 (23·5)	<0·001
Health effects			
Not purchasing sanitary pads (women only)	406 (36·4)	452 (41·0)	0·030
Not accessing needed health care/medicines	156 (8·8)	155 (8·9)	1·000
Economic effects			
Increase in food prices	1372 (77·7)	1454 (83·1)	<0·001
Complete loss of income	638 (36·2)	745 (42·7)	<0·001
Skipped a meal this week due to COVID-19	1,177 (68·0)	1·271 (73·7)	<0·001

There was no change in the proportion reporting that they were not seeking health services they needed, this was 9% in both April and May. In round three, questions also assessed measures of stigma related to COVID-19, with significantly more women than men reporting that if infected, people would stop talking to them, stop visiting their house/business, gossip about them, and treat their family badly. Women were also less likely to say people would bring them food or medicine if needed (Figure 1).

Food Security

Overall the majority of participants reported skipping a meal in the last week due to COVID-19, with some variation in the characteristics of those who skipped a meal. In fully adjusted models for April and May survey rounds, women were 6 pp more likely than men to report skipping meals in the past week due to COVID-19 (Coeff: 0·055; 95% confidence interval 0·016, 0·094) (Table 3).

For peer review only
For peer review only - http://bmjopen.bmj.com/site/about/guidelines.xhtml

Table 3: Linear regression models of factors associated with skipping meals in the last week due to COVID-19

VARIABLES	Unadjusted models Coefficients (95% CI)	Adjusted Model[1] Coefficients (95% CI)	Adjusted Model: Men Only Coefficients (95% CI)	Adjusted Model: Women only Coefficients (95% CI)
Observations		3,451	1,274	2,177
Female gender (vs male)	0·085 (0·049, 0·122)***	0·055 (0·016, 0·094)***	··	··
Age category				
18-24 years	REF	REF	REF	REF
25-34 years	0·147 (0·092, 0·202)***	0·050 (-0·013, 0·114)	-0.051 (-0.160, 0.058)	0.120 (0.040, 0.199)***
35-44 years	0·150 (0·100, 0·199)***	0·047 (-0·018, 0·112)	-0.080 (-0.201, 0.041)	0.110 (0.030, 0.190)***
45+ years	0·087 (0·032, 0·141)***	-0·002 (-0·071, 0·067)	-0.107 (-0.232, 0.018)*	0.049 (-0.037, 0.135)
Marital status				
Married	REF	REF	REF	REF
Single	-0·109 (-0·153, -0·065)***	-0·059 (-0·118, -0·000)**	-0.178 (-0.289, -0.066)***	-0.008 (-0.076, 0.061)
Divorced/ separated/ widowed	0·081 (0·039, 0·123)***	0·077 (0·031, 0·122)***	0.081 (-0.101, 0.264)	0.080 (0.032, 0.128)***
Household Size				
1-2 people	REF	REF	REF	REF
3-4 people	0·048 (-0·012, 0·108)	-0·008 (-0·069, 0·053)	0.003 (-0.086, 0.091)	-0.043 (-0.133, 0.047)
5-6 people	0·111 (0·054, 0·168)***	0·049 (-0·011, 0·110)	0.070 (-0.018, 0.159)	0.018 (-0.072, 0.107)
7+ people	0·112 (0·048, 0·176)***	0·065 (-0·001, 0·132)*	0.104 (0.005, 0.203)**	0.032 (-0.064, 0.128)
Complete loss of income due to COVID-19	0·172 (0·141, 0·203)***	0·154 (0·125, 0·184)***	0.200 (0.149, 0.251)***	0.125 (0.089, 0.162)***
Survey Round (2 vs 3)	0·057 (0·027, 0·088)***	0·049 (0·023, 0·074)***	0.048 (0.004, 0.092)**	0.051 (0.020, 0.083)***

[1]Adjusted models also control for informal settlement where participant resides
*p<0.05; ** p<0.01; *** p<0.001

Those who reported a complete loss of income were 15 pp more likely to skip a meal (Coeff: 0·154; 95% CI 0·125, 0·184). Compared with married couples, participants who were single were less likely to report skipping meals (Coeff: -0·059; 95% CI -0·118, -0·000) and those who were divorced or widowed more likely to report skipping meals (Coeff: 0·077; 95% CI 0·031, 0·122). Reportedly skipping meals due to COVID-19 also increased from April to May survey rounds; compared with April, participants in May were 5 pp more likely to report skipping a meal (Coeff: 0·049; 95% CI 0·023, 0·074).
In models stratified by gender, both men and women were more likely to skip meals if they lost employment. Among female respondents, women who are divorced, widowed, or separated were more likely to skip a meal than women who are married (Coeff: 0·080; 95% CI 0·032, 0·128). Among men, having a larger household with more members was associated with an increased probability of skipping meals.

Risk of household violence
In May, households reported more tension, arguing, violence, or fear their partner would harm them; combined these indicate increased risk of household violence. In fully adjusted models, women were 8 pp more likely to report concerns regarding household tension and violence (Coeff: 0·079; 95% CI 0·028, 0·130) (Table 4).

For peer review only - http://bmjopen.bmj.com/site/about/guidelines.xhtml

Table 4: Linear regression models of factors associated with reporting increased risk of household violence

VARIABLES	Unadjusted Models Coefficients (95% CI)	Adjusted Model[1] Coefficients (95% CI)	Men Coefficients (95% CI)	Women Coefficients (95% CI)
Observations		1,775	636	1,085
Female gender (vs male)	0·062 (0·015, 0·110)**	0·079 (0·028, 0·130)***	··	··
Age				
18-24 years	REF	REF	REF	REF
25-34 years	0·121(0·048, 0·193)***	0·034 (-0·055, 0·122)	-0.152 (-0.296, -0.008)**	0.131 (0.016, 0.245)**
35-44 years	0·081 (0·017, 0·144)**	0·015 (-0·073, 0·104)	-0.038 (-0.191, 0.114)	0.044 (-0.069, 0.156)
45+ years	0·015 (-0·054, 0·084)	-0·019 (-0·112, 0·074)	-0.106 (-0.265, 0.052)	0.026 (-0.094, 0.147)
Marital status				
Married	REF	REF	REF	REF
Single	-0·089 (-0·145, -0·034)***	-0·073 (-0·152 - 0·006)*	-0.194 (-0.336, -0.053)***	0.011 (-0.088, 0.109)
Divorced/ separated/ widowed	-0·090 (-0·152, -0·026)***	-0·106 (-0·172, -0·039)***	-0.073 (-0.314, 0.168)	-0.078 (-0.151, -0.005)**
Has skipped a meal this week due to COVID-19	0·198 (0·147, 0·250)***	0·164 (0·111, 0·218)***	0.172 (0.090, 0.254)***	0.140 (0.069, 0.212)***
Complete loss of income	0·102 (0·055, 0·148)***	0·066 (0·019, 0·113)***	0.140 (0.063, 0.218)***	0.035 (-0.025, 0.095)

[1]Adjusted models also control for informal settlement where participant resides
*p<0.05; ** p<0.01; *** p<0.001

Married couples were most likely to report this concern. Participants who had skipped a meal in the last seven days due to COVID-19 were 16 pp more likely to report increased risk of household violence (Coeff: 0·164; 95% CI 0·111, 0·218) and those who reported lost income were 7 pp more likely to report household tension and violence (Coeff: 0·066; 95% CI 0·019, 0·114). In models stratified by gender, men who had completely lost their income due to COVID-19 were 14 pp more likely to report that they experienced increased tension in the household (Coeff: 0·140; 95% CI 0·063, 0·218) while this was not significant among female respondents.

Forgoing needed health services

In fully adjusted models, women were 6 pp more likely to report not seeking health services they required in the last 2 weeks (Coeff: 0·057; 95% CI 0·037, 0·076) (Table 5).

For peer review only - http://bmjopen.bmj.com/site/about/guidelines.xhtml

Table 5: Linear regression models of factors associated with forgoing health services in the last two weeks

VARIABLES	Unadjusted Models	Adjusted Model[1]
Observations		3,455
Female gender (vs male)	0·056 (0·037, 0·076)***	0·057 (0·037, 0·076)***
Age (18-24 years REF)		
18-24 years	REF	REF
25-34 years	0·011 (-0·018, 0·041)	-0·008 (-0·042, 0·026)
35-44 years	-0·013 (-0·039, 0·103)	-0·025 (-0·053, 0·003)*
45+ years	-0·001 (-0·029, 0·027)	-0·003 (-0·034, 0·028)
Skipped a meal this week due to COVID-19	0·060 (0·040, 0·081)***	0·049 (0·030, 0·068)***
Complete loss of income	0·051 (0·032, 0·071)***	0·042 (0·020, 0·064)***
Survey Round (2 vs 3)	0·001 (-0·017, 0·020)	-0·004 (-0·022, 0·014)

[1]Adjusted models also control for informal settlement where participant resides
*p<0.05; ** p<0.01; *** p<0.001

Those who had skipped a meal due to COVID-19 were 5 pp more likely to have forgone health services (coeff: 0·049; 95% CI 0·030, 0·068) and those who had completely lost employment were also more likely to skip necessary health services (coeff: 0·042; 95% CI 0·020, 0·064). There was no significant difference between April and May. Among those that skipped health services, over half said the reason was cost (52%). Participants who skipped health services were most likely to say the service they skipped was for an acute illness (27% in April, 21% in May), followed by seeking refills for medications taken (19% in April, 20% in May).

Receiving Assistance

Between April and May survey rounds, the proportion of respondents receiving any assistance in the previous week tripled (7% in April to 21% in May); however, the proportion who had received assistance reporting that the assistance received covered their current needs decreased (47% in April, to 38% in May) (Figure 2). Most participants reported receiving soap or hand sanitizer, followed by food. When asked their single biggest need not being met, the main two items reported were food (94% in April, 86% in May) and cash (45% in April, 48% in May).

DISCUSSION

Overall, women in informal settlements in Nairobi are disproportionately affected by mitigation policies implemented to reduce the spread of COVID-19. Women are more likely to report they have skipped meals, report heightened risk of household violence, and to report forgoing necessary healthcare services due to the pandemic compared to men. Women are also more likely to report that if infected with COVID-19, they would experience stigma and have less social support. Between April and May, the proportion of respondents reporting that they are receiving government or NGO assistance has increased, but the proportion reporting that this assistance is enough to meet their basic needs has decreased. Our findings suggest that three months into the pandemic response, households in urban informal settlements in Nairobi are already experiencing significant economic, social and health related harms. Increasing coverage of government assistance in the form of cash transfers or food distributions is critical, along with increasing safe and affordable access to needed health services to prevent secondary outbreaks or

For peer review only - http://bmjopen.bmj.com/site/about/guidelines.xhtml

long-term health problems. This will also help people sustain COVID-19 prevention measures over the longer term, for example, enabling them to stay home if they have enough food. These steps will ensure that progress toward development goals to date are not lost or reversed. Attention to the needs of women in these settings is critical as they are disproportionately vulnerable. Our findings are likely to be broadly applicable to many other urban African contexts where there are large urban slums and strict mitigation policies.

Our study identified the serious threat of food insecurity three months into the pandemic response in Nairobi, with almost three quarters of all study participants reporting they skipped a meal in the previous week due to COVID-19. In crisis settings, women and children disproportionately bear the brunt of the effects of food insecurity with both short- and long-term implications for health and well-being.[12] Due to pandemic mitigation policies and movement restrictions, county government entities across Kenya have begun to close local, informal, outdoor markets where a majority of food is distributed.[27] These disruptions are likely to be causing supply shortages and price increases including in urban areas and reportedly making it difficult to access food, especially for families with lost unemployment.[12,27] An African Union report projects that up to 20 million jobs could be lost in the region due to COVID-19.[28] Our study found that divorced women are at a higher risk of food insecurity compared to married and single women. This is perhaps due to the challenges a divorced woman faces as the single earner in the household while still is also responsible for taking care of children.[29,30] Further research should explore this connection.

Our study found that overall households are experiencing increased risk of household violence. This may be related to forced time spent in the home, widespread and sudden loss of employment leading to increased tension, and limited access to gender-based violence support programs. During COVID-19 lockdowns in China, domestic violence reports tripled as people were forced to stay home[19,31] During Ebola, gender based violence support initiatives were defunded due to prioritization of infection control, while in South Africa, rumors about COVID-19 led to a drop in utilization of gender-based violence support programs.[31,32] Multiple studies have found that intimate partner violence risk increases when employment is lost, including if women earn more than their partners or during socioeconomic hardship due to conflict.[33–35] This supports our finding that male respondents reported more household tension, arguing and household violence risk if they had lost employment due to COVID-19. Our findings also indicate that increased food insecurity might be associated with increased risk of violence; this connection is supported by studies in South Africa and Ethiopia that found a significant link between household food insecurity status and increased risk of violence.[36,37] As this pandemic continues, it will be critical to ensure access to mental health and gender-based violence support for the most vulnerable, particularly women and their children, as unemployment and challenges accessing food and other resources may increase with prolonged physical distancing policy.

In addition to food insecurity and household violence, there is concern that COVID-19 will lead to adverse secondary health effects if routine care including immunization, nutrition, sexual and reproductive health, and antenatal care are not utilized. The main reason respondents gave for skipping a necessary health service was that they could not afford the service. People may not be able to afford necessary care due to unemployment and rising costs of food and other basic necessities. In 2013, the Kenyan Ministry of Health reported that 83 percent of Kenyans were not financially able to cover healthcare costs and 1.5 million Kenyans are driven into poverty each year because of this.[38] The economic insecurity that will result from COVID-19 will likely reverse any progress on this issue, causing potential outbreaks of other diseases and worsening health outcomes. Respondents also cited to a lesser degree concerns regarding stigma and access challenges. Participants in the survey report clinics are reducing patient volume to reduce risk of infection. On the demand side, people may avoid seeking care if they are concerned they will get the disease, or that they will be forced to quarantine. According to the World Health Organization, people who fear stigma related to COVID-19 may be driven to hide their illness, delay seeking healthcare, and be less likely to adopt healthy behaviors.[39] During Ebola, studies found that unprepared health systems heightened this mistrust of health systems in the community and kept women from accessing maternal and child health services.[40,41] A study found only 20 to 30

For peer review only - http://bmjopen.bmj.com/site/about/guidelines.xhtml

percent of urban residents seeking formal healthcare were able to access it, and previously increasing trends in MCH utilization sharply declined, due to mistrust in the health system,[42] and as of 2017 have yet to return to pre-Ebola levels.[43,44] Women in our study report forgoing critical health services for themselves and for their children, signaling the potential for secondary adverse health effects. The government and other stakeholders must prioritize ensuring affordable and accessible health services in a healthcare setting that is safe for providers and patients.

This study has some limitations. First, we sampled from participants from AGI-K and NISITU cohorts, meaning that the COVID-19 cohort sample is representative of households in which an adolescent resides, but not of all households. A household with only an elderly person or only early primary school aged children, for example, would not have been eligible for inclusion. Second, all of the questions are self-reported, so there may be some bias in how participants respond to questions or recall bias. Relatedly, we interviewed one adult per household, so their responses may not reflect all views or experiences of every household member. Other challenges relate to the phone-based nature of this survey. Since this was a phone-based survey, we faced some challenges verifying the respondent and had to rely on their responses. There also may have been some bias from who we could reach by phone, as some phone numbers never picked up. Another potential challenge with phone-based data collection is privacy; respondents may not have answered each question honestly if they were at home with others nearby. We tried to frame sensitive questions as yes/no responses so that respondents could answer honestly without others hearing the topic.

CONCLUSION

Our findings suggest that overall, three months into the COVID-19 pandemic and mitigation response, households in informal settlements are facing severe adverse social and economic effects, with women disproportionately impacted. Governments around the world are taking steps to balance the risks of COVID-19 transmission against the severe economic and social toll that will result from prolonged economic disruption. In the short-term, our findings highlight the urgent need to address rising unemployment, food insecurity and to strengthen existing structures at the community level to address gender-based violence. Social safety net programs or food distributions may be required as stop gap measures until COVID-19 is under control in Kenya. Future research and policy should address the long-term effects of this disruptive event, to understand how the pandemic affected the life trajectories of urban residents of informal slums and to take steps to ensure that households do not fall into poverty and extreme poverty as a result. Taking these steps in the short and long term are critical to protect the most vulnerable households in Kenya, preserve development gains made to date and sustain a positive course in the long-term.

Funding: UK Department for International Development through Innovations for Poverty Action Peace & Recovery COVID-19 rapid response grant.

Competing Interests: None

Acknowledgements: the authors would like to acknowledge all of the work done by our field team to collect these surveys.

Author contributions: JP helped develop the study design, instrument, conducted data analysis and led drafting of the manuscript. KA led the study design, implementation, and supported with developing the analysis plan and provided input on the manuscript. NR conducted the literature review, supported with data analysis, and supported drafting of the manuscript. TA, BK, and RO supported study design, implementation, and provided input on the manuscript. JBT supported with study design, data management and analysis, and input on the manuscript. DM, EM, FM, and MN supported with study design, data collection, field team supervision, and provided input the manuscript. TDN conceived of the study, helped develop the study design, analysis plan, and provided input on the manuscript.

For peer review only - http://bmjopen.bmj.com/site/about/guidelines.xhtml

REFERENCES

1 Cucinotta D, Vanelli M. WHO declares COVID-19 a pandemic. *Acta Biomed.* 2020. doi:10.23750/abm.v91i1.9397

2 World Health Organization. New WHO estimates: Up to 190 000 people could die of COVID-19 in Africa if not controlled. WHO Africa. 2020.https://www.afro.who.int/news/new-who-estimates-190-000-people-could-die-covid-19-africa-if-not-controlled

3 Quaife M, Zandvoort K van, Gimma A, *et al.* The impact of COVID-19 control measures on social contacts and transmission in Kenyan informal settlements. *medRxiv* Published Online First: 2020. doi:10.1101/2020.06.06.20122689

4 Harrisburg. K. Half urban population of sub-Saharan Africa still living in slums: study. Reuters. https://www.reuters.com/article/us-africa-housing-disease/half-urban-population-of-sub-saharan-africa-still-living-in-slums-study-idUSKCN1R823K

5 Snyder RE, Marlow MA, Riley LW. Ebola in urban slums: The elephant in the room. *Lancet Glob Heal.* 2014. doi:10.1016/S2214-109X(14)70339-0

6 Mehtar S, Preiser W, Lakhe NA, *et al.* Limiting the spread of COVID-19 in Africa: one size mitigation strategies do not fit all countries. *Lancet Glob. Heal.* 2020. doi:10.1016/S2214-109X(20)30212-6

7 Kabale N. Coronavirus: Kenya takes bold steps after cases rise to three. Dly. Nation. https://www.nation.co.ke/dailynation/news/coronavirus-kenya-takes-bold-steps-after-cases-rise-to-three-278692

8 Center. JHC-19 R. COVID-19 Case Tracker 2020. Available from https//coronavirus.jhu.edu/.

9 UN Habitat. Kenya Habitat Country Programme Document. (2018-2021). Nairobi, Kenya.: 2019. https://unhabitat.org/sites/default/files/2019/09/hcpd_kenya_2018_-_2021_0.pdf

10 Austrian K, Pinchoff J, Tidwell JB, *et al.* COVID-19 Related Knowledge, Attitudes, Practices and Needs of Households in Informal Settlements in Nairobi, Kenya. *SSRN Electron J* Published Online First: 2020. doi:10.2139/ssrn.3576785

11 Roberton T, Carter ED, Chou VB, *et al.* Early estimates of the indirect effects of the COVID-19 pandemic on maternal and child mortality in low-income and middle-income countries: a modelling study. *Lancet Glob Heal.* Published Online First: 2020. doi:10.1016/s2214-109x(20)30229-1

12 The Lancet Global Health. Food insecurity will be the sting in the tail of COVID-19. *Lancet Glob Heal*;**Volume 8.**

13 Ruel MT, Garrett JL, Hawkes C, *et al.* The Food, Fuel, and Financial Crises Affect the Urban and Rural Poor Disproportionately: A Review of the Evidence. *J Nutr* Published Online First: 2010. doi:10.3945/jn.109.110791

14 World Health Organization. 2019 Novel Coronavirus Strategic Preparedness and Response Plan. 2020. https://www.who.int/publications/i/item/strategic-preparedness-and-response-plan-for-the-new-coronavirus

15 Corburn J, Vlahov D, Mberu B, *et al.* Slum Health: Arresting COVID-19 and Improving Well-Being in Urban Informal Settlements. *J Urban Heal* Published Online First: 2020. doi:10.1007/s11524-020-00438-6

16 Wenham C, Smith J, Morgan R. COVID-19: the gendered impacts of the outbreak. *Lancet.* 2020. doi:10.1016/S0140-6736(20)30526-2

17 Lucas B. Impacts of Covid-19 on inclusive economic growth in middle-income countries. *KD4 Help Rep 811 Bright UK Inst Dev Stud*

18 van Gelder N, Peterman A, Potts A, *et al.* COVID-19: Reducing the risk of infection might increase the risk of intimate partner violence. *EClinicalMedicine.* 2020. doi:10.1016/j.eclinm.2020.100348

19 World Health Organization. COVID-19 and violence against women: What the health sector / system can do. 2020. https://apps.who.int/iris/handle/10665/331699

20 Joska JA, Andersen L, Rabie S, *et al.* COVID-19: Increased Risk to the Mental Health and Safety

For peer review only - http://bmjopen.bmj.com/site/about/guidelines.xhtml

of Women Living with HIV in South Africa. *AIDS Behav.* 2020. doi:10.1007/s10461-020-02897-z
21 Elston JWT, Cartwright C, Ndumbi P, *et al.* The health impact of the 2014–15 Ebola outbreak. *Public Health.* 2017. doi:10.1016/j.puhe.2016.10.020
22 Strong A.E. SDA. Effects of the West African Ebola Epidemic on Health Care of Pregnant Women: Stigmatization With and Without Infection. *Springer, Cham.*
23 World Vision. Covid-19 Aftershocks: Secondary Impacts Threaten More Children'S Lives Than Disease Itself. 2020. https://www.wvi.org/publications/report/coronavirus-health-crisis/covid-19-aftershocks-secondary-impacts-threaten-more.
24 Colavita F, Biava M, Castilletti C, *et al.* Measles cases during ebola outbreak, West Africa, 2013–2106. *Emerg. Infect. Dis.* 2017. doi:10.3201/eid2306.161682
25 Eggers R. WHO Kenya Coronavirus update. *World Heal Organ.*
26 Austrian K, Muthengi E, Mumah J, *et al.* The Adolescent Girls Initiative-Kenya (AGI-K): Study protocol. *BMC Public Health* Published Online First: 2016. doi:10.1186/s12889-016-2888-1
27 Kusmer A. Coronavirus — and locusts — threaten Kenya's food security. The World. https://www.pri.org/stories/2020-05-13/coronavirus-and-locusts-threaten-kenya-s-food-security
28 African Union. Impact of the Coronavirus (COVID-19) on the African Economy. 2020. https://icsb.org/wp-content/uploads/2020/04/Covid-19-impact-on-African-economy.pdf.pdf.pdf.pdf
29 Grant MJ, Pike I. Divorce, living arrangements, and material well-being during the transition to adulthood in rural Malawi. *Popul Stud (NY)* Published Online First: 2019. doi:10.1080/00324728.2018.1545919
30 Lawson DW, Schaffnit SB, Hassan A, *et al.* Father absence but not fosterage predicts food insecurity, relative poverty, and poor child health in northern Tanzania. *Am J Hum Biol* Published Online First: 2017. doi:10.1002/ajhb.22938
31 Gould C. Gender-Based Violence During Lockdown - Looking for Answers [analysis]. Mark. Watch. https://www.marketwatch.com/press-release/gender-based-violence-during-lockdown---looking-for-answers-analysis-2020-05-12
32 John N, Casey SE, Carino G, *et al.* Lessons Never Learned: Crisis and gender-based violence. *Dev World Bioeth* Published Online First: 2020. doi:10.1111/dewb.12261
33 Abramsky T, Lees S, Stöckl H, *et al.* Women's income and risk of intimate partner violence: Secondary findings from the MAISHA cluster randomised trial in North-Western Tanzania. *BMC Public Health.* Published Online First: 2019. doi:10.1186/s12889-019-7454-1
34 Rees S, Mohsin M, Tay AK, *et al.* Risk of perpetrating intimate partner violence amongst men exposed to torture in conflict-affected Timor-Leste. *Glob Ment Heal.* Published Online First: 2018. doi:10.1017/gmh.2018.16
35 Cardoso LF, Gupta J, Shuman S, *et al.* What Factors Contribute to Intimate Partner Violence Against Women in Urban, Conflict-Affected Settings? Qualitative Findings from Abidjan, Côte d'Ivoire. *J Urban Heal.* Published Online First: 2016. doi:10.1007/s11524-016-0029-x
36 Hatcher AM, Stöckl H, McBride RS, *et al.* Pathways From Food Insecurity to Intimate Partner Violence Perpetration Among Peri-Urban Men in South Africa. *Am J Prev Med.* Published Online First: 2019. doi:10.1016/j.amepre.2018.12.013
37 Andarge E, Shiferaw Y. Disparities in intimate partner violence among currently married women from food secure and insecure urban households in South Ethiopia: A community based comparative cross-sectional study. *Biomed Res Int.* Published Online First: 2018. doi:10.1155/2018/4738527
38 Ministry of Health. Nairobi: Government of Kenya. The Kenya household health expenditure and utilisation survey. 2013. https://www.healthpolicyproject.com/pubs/745_KHHUESReportJanuary.pdf
39 World Health Organization. Social Stigma associated with COVID-19. 2020. https://www.who.int/docs/default-source/coronaviruse/covid19-stigma-guide.pdf
40 Jones S, Sam B, Bull F, *et al.* 'Even when you are afraid, you stay': Provision of maternity care

For peer review only - http://bmjopen.bmj.com/site/about/guidelines.xhtml

during the Ebola virus epidemic: A qualitative study. *Midwifery.* Published Online First: 2017. doi:10.1016/j.midw.2017.05.009

41 Woskie LR, Fallah MP. Overcoming distrust to deliver universal health coverage: Lessons from Ebola. *BMJ.* Published Online First: 2019. doi:10.1136/bmj.l5482

42 Nuriddin A, Jalloh MF, Meyer E, *et al.* Trust, fear, stigma and disruptions: Community perceptions and experiences during periods of low but ongoing transmission of Ebola virus disease in Sierra Leone, 2015. *BMJ Glob Heal.* Published Online First: 2018. doi:10.1136/bmjgh-2017-000410

43 McQuilkin PA, Udhayashankar K, Niescierenko M, *et al.* Health-care access during the Ebola virus epidemic in Liberia. *Am J Trop Med Hyg.* Published Online First: 2017. doi:10.4269/ajtmh.16-0702

44 Delamou A, Ayadi AME, Sidibe S, *et al.* Effect of Ebola virus disease on maternal and child health services in Guinea: a retrospective observational cohort study. *Lancet Glob Heal.* Published Online First: 2017. doi:10.1016/S2214-109X(17)30078-5

For peer review only

For peer review only - http://bmjopen.bmj.com/site/about/guidelines.xhtml

Figure 1: Proportion of participants reporting stigma and social support related to COVID-19 by gender as measured in May (Round 3).

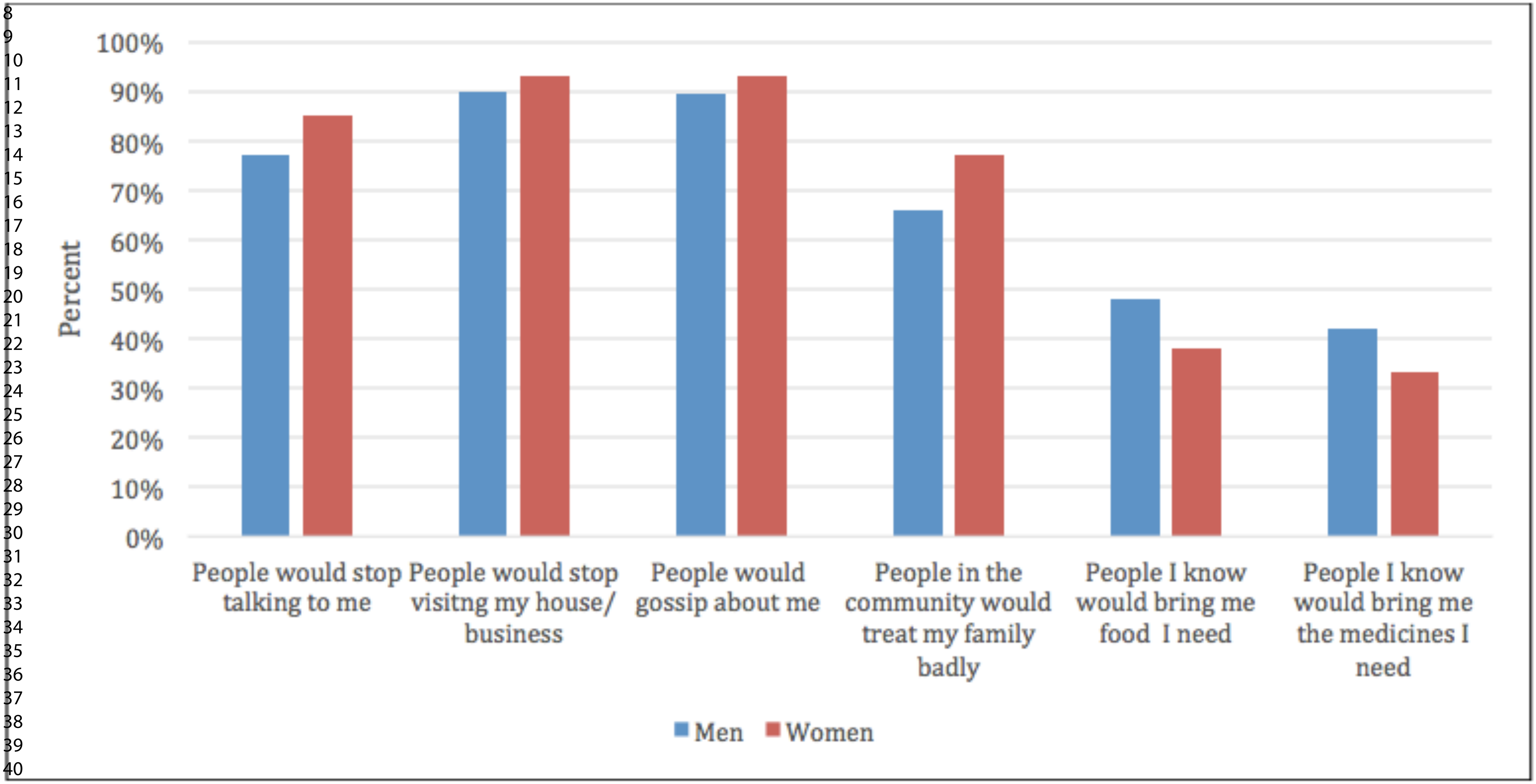

Figure 2: Proportion of participants reporting assistance received and specific types of support received, comparing April (Round 2) to May (Round 3)

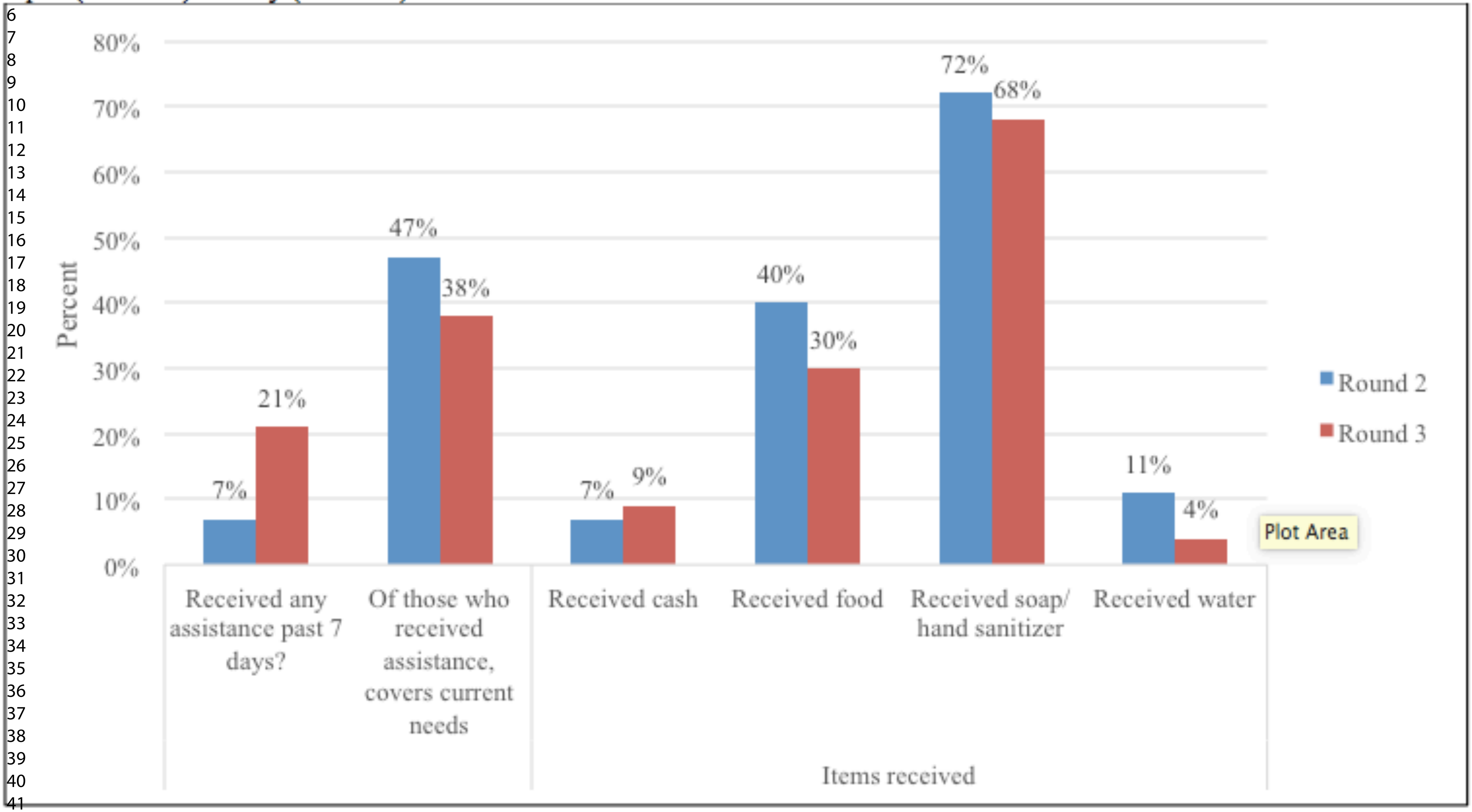

STROBE Statement—Checklist of items that should be included in reports of ***cohort studies***

	Item No	Recommendation	Page No
**Title and abstract**	1	(*a*) Indicate the study's design with a commonly used term in the title or the abstract	1
		(*b*) Provide in the abstract an informative and balanced summary of what was done and what was found	2
**Introduction**			
Background/rationale	2	Explain the scientific background and rationale for the investigation being reported	3-4
Objectives	3	State specific objectives, including any prespecified hypotheses	4
**Methods**			
Study design	4	Present key elements of study design early in the paper	4
Setting	5	Describe the setting, locations, and relevant dates, including periods of recruitment, exposure, follow-up, and data collection	4
Participants	6	(*a*) Give the eligibility criteria, and the sources and methods of selection of participants. Describe methods of follow-up	4-5
		(*b*) For matched studies, give matching criteria and number of exposed and unexposed	-
Variables	7	Clearly define all outcomes, exposures, predictors, potential confounders, and effect modifiers. Give diagnostic criteria, if applicable	5
Data sources/ measurement	8*	For each variable of interest, give sources of data and details of methods of assessment (measurement). Describe comparability of assessment methods if there is more than one group	5
Bias	9	Describe any efforts to address potential sources of bias	5
Study size	10	Explain how the study size was arrived at	4
Quantitative variables	11	Explain how quantitative variables were handled in the analyses. If applicable, describe which groupings were chosen and why	5
Statistical methods	12	(*a*) Describe all statistical methods, including those used to control for confounding	
		(*b*) Describe any methods used to examine subgroups and interactions	5
		(*c*) Explain how missing data were addressed	
		(*d*) If applicable, explain how loss to follow-up was addressed	
		(*e*) Describe any sensitivity analyses	
**Results**			
Participants	13*	(a) Report numbers of individuals at each stage of study—eg numbers potentially eligible, examined for eligibility, confirmed eligible, included in the study, completing follow-up, and analysed	6
		(b) Give reasons for non-participation at each stage	
		(c) Consider use of a flow diagram	
Descriptive data	14*	(a) Give characteristics of study participants (eg demographic, clinical, social) and information on exposures and potential confounders	6-7
		(b) Indicate number of participants with missing data for each variable of interest	
		(c) Summarise follow-up time (eg, average and total amount)	
Outcome data	15*	Report numbers of outcome events or summary measures over time	

For peer review only

For peer review only - http://bmjopen.bmj.com/site/about/guidelines.xhtml

Main results	16	(*a*) Give unadjusted estimates and, if applicable, confounder-adjusted estimates and their precision (eg, 95% confidence interval). Make clear which confounders were adjusted for and why they were included<br>(*b*) Report category boundaries when continuous variables were categorized<br>(*c*) If relevant, consider translating estimates of relative risk into absolute risk for a meaningful time period	7-10
Other analyses	17	Report other analyses done—eg analyses of subgroups and interactions, and sensitivity analyses	
**Discussion**			
Key results	18	Summarise key results with reference to study objectives	10-12
Limitations	19	Discuss limitations of the study, taking into account sources of potential bias or imprecision. Discuss both direction and magnitude of any potential bias	12
Interpretation	20	Give a cautious overall interpretation of results considering objectives, limitations, multiplicity of analyses, results from similar studies, and other relevant evidence	12
Generalisability	21	Discuss the generalisability (external validity) of the study results	12
**Other information**			
Funding	22	Give the source of funding and the role of the funders for the present study and, if applicable, for the original study on which the present article is based	12

*Give information separately for exposed and unexposed groups.

Note: An Explanation and Elaboration article discusses each checklist item and gives methodological background and published examples of transparent reporting. The STROBE checklist is best used in conjunction with this article (freely available on the Web sites of PLoS Medicine at http://www.plosmedicine.org/, Annals of Internal Medicine at http://www.annals.org/, and Epidemiology at http://www.epidem.com/). Information on the STROBE Initiative is available at http://www.strobe-statement.org.

For peer review only - http://bmjopen.bmj.com/site/about/guidelines.xhtml

BMJ Open

Gendered economic, social, and health effects of the COVID-19 pandemic and mitigation policies in Kenya: evidence from a prospective cohort survey in Nairobi informal settlements

Journal:	*BMJ Open*
Manuscript ID	bmjopen-2020-042749.R1
Article Type:	Original research
Date Submitted by the Author:	04-Nov-2020
Complete List of Authors:	Pinchoff, Jessie; Population Council, Poverty, Gender and Youth<br>Austrian, Karen; Population Council, Poverty, Gender & Youth<br>Rajshekhar, Nandita; Independent consultant<br>Abuya, Timothy; Populat Council Gen Accid Insurance House, Re; Population Council Kenya,<br>Kangwana, Beth; Population Council, Poverty, Gender & Youth<br>Ochako, Rhoune; Population Council, Poverty, Gender & Youth<br>Tidwell, James; World Vision International,<br>Mwanga, Daniel; Population Council, Poverty, Gender & Youth<br>Muluve, Eva; Population Council, Poverty, Gender & Youth<br>Mbushi, Faith; Population Council, Poverty, Gender & Youth<br>Nzioki, Mercy; Population Council, Poverty, Gender & Youth<br>Ngo, Thoai; Population Council, Poverty, Gender and Youth
**Primary Subject Heading**:	Global health
Secondary Subject Heading:	Epidemiology, Infectious diseases, Public health
Keywords:	Epidemiology < TROPICAL MEDICINE, Public health < INFECTIOUS DISEASES, PUBLIC HEALTH

SCHOLARONE™
Manuscripts

I, the Submitting Author has the right to grant and does grant on behalf of all authors of the Work (as defined in the below author licence), an exclusive licence and/or a non-exclusive licence for contributions from authors who are: i) UK Crown employees; ii) where BMJ has agreed a CC-BY licence shall apply, and/or iii) in accordance with the terms applicable for US Federal Government officers or employees acting as part of their official duties; on a worldwide, perpetual, irrevocable, royalty-free basis to BMJ Publishing Group Ltd ("BMJ") its licensees and where the relevant Journal is co-owned by BMJ to the co-owners of the Journal, to publish the Work in this journal and any other BMJ products and to exploit all rights, as set out in our licence.

The Submitting Author accepts and understands that any supply made under these terms is made by BMJ to the Submitting Author unless you are acting as an employee on behalf of your employer or a postgraduate student of an affiliated institution which is paying any applicable article publishing charge ("APC") for Open Access articles. Where the Submitting Author wishes to make the Work available on an Open Access basis (and intends to pay the relevant APC), the terms of reuse of such Open Access shall be governed by a Creative Commons licence – details of these licences and which Creative Commons licence will apply to this Work are set out in our licence referred to above.

Other than as permitted in any relevant BMJ Author's Self Archiving Policies, I confirm this Work has not been accepted for publication elsewhere, is not being considered for publication elsewhere and does not duplicate material already published. I confirm all authors consent to publication of this Work and authorise the granting of this licence.

For peer review only

Title: Gendered economic, social, and health effects of the COVID-19 pandemic and mitigation policies in Kenya: evidence from a prospective cohort survey in Nairobi informal settlements

Authors: Jessie Pinchoff PhD[1], Karen Austrian PhD[2], Nandita Rajshekhar MPH[3], Timothy Abuya PhD[2], Beth Kangwana PhD[2], Rhoune Ochako PhD[2], James B Tidwell PhD[4,5], Daniel Mwanga[2], Eva Muluve[2], Faith Mbushi[2], Mercy Nzioki[2], Thoai D Ngo PhD[1]

Author Information:

[1] Population Council, Department of Poverty, Gender and Youth, New York NY 10017
[2] Population Council – Kenya, Nairobi Kenya
[3] Independent consultant
[4] Harvard Kennedy School of Government, Cambridge, MA
[5] World Vision, Washington, DC

Corresponding Author:
Jessie Pinchoff, PhD
Population Council
Department of Poverty, Gender and Youth
One Dag Hammarskjold Plaza #3
New York, NY 10017
Email: jpinchoff@popcouncil.org

For peer review only

ABSTRACT

Objectives: COVID-19 may spread rapidly in densely populated urban informal settlements. Kenya swiftly implemented mitigation policies; we assess the economic, social, and health related harm disproportionately impacting women.
Design: A prospective longitudinal cohort study with repeated mobile-phone surveys in April, May and June, 2020.
Participants and Setting: 2,009 households across five informal settlements in Nairobi, sampled from two previously interviewed cohorts.
Primary and Secondary Outcome Measures: Outcomes include food insecurity, risk of household violence, and forgoing necessary health services due to the pandemic. Gender stratified linear probability regression models were constructed to determine factors associated with these outcomes.
Results: By May, more women than men reported adverse effects of COVID-19 mitigation policies on their lives. Women were 6 percentage points (pp) more likely to skip a meal vs men (coeff: 0·055; 95% Confidence Interval (CI) 0·016, 0·094), and those who had completely lost their income were 15 pp more likely vs those employed (coeff: 0·154; 95% CI 0·125, 0·184) to skip a meal. Compared to men, women were 8 pp more likely to report increased risk of household violence (coeff: 0·079; 95% CI 0·028, 0·130), and 6 pp more likely to forgo necessary healthcare (coeff: 0·057; 95% CI 0·038, 0·076).
Conclusions: The pandemic rapidly and disproportionately impacted the lives of women. As Kenya reopens, policymakers must deploy assistance to ensure women in urban slums are able to return to work, get healthcare and services they need to not lose progress on gender equity made to date.

Key Words: COVID-19, gender, Kenya, urban, health, epidemiology

ARTICLE SUMMARY

Strengths and Limitations of this Study

- This is one of the first longitudinal cohort studies on COVID-19 vulnerability and risks in informal settlements in a sub-Saharan African city.
- These findings highlight the disproportionate risks and impacts shouldered by women in the COVID-19 pandemic, and identify key characteristics associated with food insecurity, risk of household violence, and forgoing necessary medical care by gender. By doing this study in partnership with government actors involved in the COVID-19 response in Kenya, these results will inform social support programs in response to this pandemic to ensure the most vulnerable receive targeted assistance, with attention to the needs of women.
- There are two limitations: first, since participants of this study were sampled from existing cohorts from ongoing adolescent studies, the study population may not be fully representative of the setting population. Households were only eligible if an adolescent resided in the home; therefore, some households are not represented. Second, the survey was conducted among those we were able to reach by mobile phone, and responses are self-reported, which can lead to reporting and selection bias.

For peer review only

For peer review only - http://bmjopen.bmj.com/site/about/guidelines.xhtml

INTRODUCTION

COVID-19, a highly transmissible respiratory disease caused by the novel severe acute respiratory syndrome coronavirus 2 (SARS-CoV2), was officially declared a pandemic on March 11th 2020 by the World Health Organization (WHO).[1] Despite the lower than expected transmission to date, there is potential for a high number of cases and COVID-19 deaths in Sub-Saharan Africa if containment and mitigation efforts fail.[2] Sub-Saharan Africa has rapidly urbanized; 47% of urban residents in the region live in informal settlements that are ill equipped to handle a disease outbreak.[3,4] These areas face increased risk for rapid spread of coronavirus due to high population density, inadequate housing and sanitation facilities, and intense levels of social mixing.[5] Coupled with the higher risks of disease transmission, residents of informal settlements face the concurrent shock of devastating impacts from physical distancing policies and lockdowns, including closing of businesses, schools, and ban on large gatherings. Mitigation policies may slow the transmission of the virus, but they come with a heavy social and economic toll on poor urban populations, potentially higher for women.[6] There has been extensive theorizing regarding how COVID-19 will impact African populations to date, but little systematic research. This study is one of the first prospective cohort studies conducted during COVID-19 to understand the experiences of those residing in urban informal settlements, including a broad set of indicators and measures across sectors and disciplines to holistically understand the interconnected short- and long-term effects of the pandemic.

In Kenya, the first case of COVID-19 was detected on March 13th and resulted in the Kenyan government directing the immediate closure of schools and restaurants/bars and the prohibition of large gatherings. Two weeks later, on March 26th Kenya banned international flights.[7] On April 6th, the Nairobi Metropolitan Area and three counties in coastal Kenya were contained, restricting movement into and out of these counties. Many businesses and stores closed as a result. Three months into the crisis, Kenya has confirmed 6,673 cases and 149 deaths related to COVID-19 (as of July 1st, 2020).[8] An estimated 60-70% of Nairobi's more than 4 million residents reside in urban informal settlements or slums.[9] From March 30th-31st 2020, Population Council's COVID-19 mobile phone-based survey among 2,009 informant settlement dwellers found 61% of respondents reported physical distancing measures would be challenging to follow, as it would risk their income.[10] The survey also found about one in five were worried about food shortages (22%) and about one in three were worried about job or income loss (34%).[10] Lack of income may be a significant challenge if prices for food and other critical needs go up, as news outlets are reporting. Recent reports express concerns that COVID-19 and Kenya's mitigation policies may lead to severe setbacks in access to healthcare, nutrition, immunization, other diseases, and gender equity.[11,12]

Urban poor households are highly vulnerable during crises, and when faced with severe external shocks, are less able to cope with the health and financial impacts, as seen in previous case studies.[13] Implementing physical distancing and personal hygiene measures recommended by the World Health Organization[14] to curb the spread of the outbreak will be challenging if not impossible in urban slums.[15] Those who reside in slums are less likely to have access to potable water or a latrine in their home; in the Kibera slum in Nairobi, residents have one latrine for 50-150 people.[16] While there is fear regarding the spread of COVID-19, the potential harm caused by income loss, food insecurity, and foregoing health services may be just as severe, particularly for women.[17] Women are less likely to employed in informal settlements, and employment they do have is often tenuous and informal even before COVID-19. One study found only 22% of adult men and 3.6% of adult women report salaried employment in Nairobi slums.[18] Women also disproportionately take on unpaid care burdens of children and the elderly, potentially forcing them to exit the workforce. This may leave women and female headed households particularly vulnerable or dependent on male partners. Experts have yet to determine the severity of economic and social impacts likely to result from disease prevention and containment methods. A holistic approach, meaning one that takes into account the simultaneous impacts on income, access to health services and experience of violence, and the linkages between them, is needed to understand these interrelated secondary effects.

For peer review only - http://bmjopen.bmj.com/site/about/guidelines.xhtml

During and after emergencies, conflicts, or epidemics, women often face extreme challenges due to the gender inequality and discrimination that existed before and is exacerbated due to sudden shifts in gender roles and relations.[19] Studies have found that women are more likely than men to face increased insecurity, restricted mobility, and other major challenges.[19] During the Ebola outbreak in Sierra Leone, women disproportionately lost their jobs compared to men and the related economic disruptions impacted girls' education with more girls being pressured to drop out over boys due to economic constraints.[20] Women also tend to take on more unpaid household labor such as cooking, cleaning, and childcare during times of crises including COVID-19.[21] With physical distancing, there are concerns that gender-based violence (GBV) may increase. Distancing may lead to social isolation and reduce the safety of victims. Stress and coping mechanisms such as increased alcohol consumption may also lead to more instances of violence.[22] In China, reports of domestic violence tripled during lockdown,[23] while in South Africa, reports have increased with 87,000 reports of domestic violence were recorded in the first week of lockdown alone, despite the ban on alcohol.[24,25] At the same time, services to support survivors are being disrupted.[21] These trends threaten the progress made toward gender equality and GBV reduction efforts.

Access to healthcare is another critical dimension that may be exacerbated by the pandemic for women in particular. People may not be able to afford healthcare due to unemployment caused by the pandemic, facilities may restrict patient volume to minimize infection risk, and even if healthcare is available, people may avoid seeking care due to fear of infection at clinics. While some preliminary studies show men may be more likely to die of COVID-19,[26–29] women are adversely impacted in other ways. Mobility restrictions and the cost of healthcare fees may disproportionately limit the ability of women to seek healthcare, impacting their health but also the health of children in their care. A recent report suggests 30 million children's lives may be at risk if secondary effects on health systems are similar for COVID-19 as they were for Ebola.[30] For example, shortly after Ebola there was a rise in measles cases, due to the drop in vaccinations caused by the crisis.[31] There Is already the potential for this pattern in Kenya where already, compared with 2018 and 2019, under-5 OPD attendance and vaccination rates are significantly down.[29] Diverting resources to emerging threats can lead to neglecting other infectious diseases resulting in new waves of disease outbreaks and many lives lost, or shifting priorities that may make it more difficult to access sexual and reproductive health services.[21] These lessons need to be considered when allocating resources for COVID-19 and instating containment policies.

The primary objective of this study is to assess the short-term economic, social, and health effects of COVID-19 and related mitigation measures among a prospective, longitudinal cohort of households sampled from five of Nairobi's informal settlements, with a focus on disproportionate burden placed on women. This cohort study was conducted rapidly during the initial COVID-19 response in Kenya, developed with the aim of understanding the experiences of households and the dynamic effects of the pandemic as the situation evolves. We evaluated disparities by gender, as research from previous health and humanitarian crises suggest women are likely to be disproportionately impacted. Our findings will help develop and better target both short- and long-term policy and interventions. It will also highlight the prevalence of gender inequity and how this may impact the trajectories of women's lives.

METHODS

The Population Council, in collaboration with the Kenyan Ministry of Health, has conducted mobile phone surveys across five urban slums in Nairobi (Kibera, Mathare, Kariobangi, Dandora, and Huruma). The study methods have been described elsewhere.[10] The study participants were randomly sampled from two existing longitudinal cohort studies, the Adolescent Girls Initiative-Kenya (AGI-K) and NISITU. Prior to COVID-19, the AGI-K urban cohort in Kibera and Huruma repeatedly sampled 2,565 randomly selected households that had at least one adolescent resident. This cohort was part of a four-arm randomized controlled trial testing the impact of adolescent girls programs. This included a baseline data collection in 2015, a second round of data collection in 2017, and a third round in 2019.[32] The NISITU

cohort consisted of 4,519 households randomly sampled from households in Kariobangi, Dandora, and Mathare informal settlements. NISITU is a quasi-experimental study evaluating the effects of a gender transformative program for girls, boys, and young men. The NISITU baseline was conducted in early 2018 and the second wave of data collection in late 2019. NISITU and AGI-K mainly served as a sampling frame for the COVID-19 monthly surveys. For both cohorts, an initial household listing was conducted in the study sites to create a sampling frame of households with an eligible adolescent for the study. The last round of data collection for each was recently completed (September 2019 for AGI-K and in January 2020 for NISITU), therefore phone numbers for each head of household were up to date. All households were eligible for inclusion as long as an adult was reached on the phone. We randomly sampled from all 7,500 households with available phone numbers (if multiple numbers were available, one was randomly selected), stratified by informal settlement. For the first round, we estimated a minimum of 400 participants per site was required at baseline (+/-5% confidence interval calculation from a conservative 50% prevalence estimate for COVID-19 related knowledge). As the sample was randomly drawn from the pool of phone numbers, and there was no randomization of intervention at this stage, no design effect of the study was considered. The COVID-19 cohort consists of about 2,009 adult household members interviewed on March 30th-31st (round 1). We re-interviewed 1,768 of these on April 13-14th (round 2) and 1,750 on May 10-11th 2020 (round 3). Due to ongoing physical distancing policies in Kenya, all surveys were conducted on the phone to protect both participants and surveyors from potential COVID-19 spread. Each survey lasted about 30 minutes, with some questions asked across all three surveys and some unique questions in each of the three surveys.

Survey Instruments

The COVID-19 questionnaires used adapted standardized questions wherever possible, such as questions from the Demographic and Health Survey and the WHO/UNICEF Joint Monitoring Program on water and sanitation. A total of 77 Kenyan surveyors were trained on conducting mobile phone surveys, and before each survey round a training session was held to review the new questions. The first round questionnaire included questions on basic demographics, awareness of COVID-19, knowledge, perceived risk, preventive behaviors being implemented, channels of information, and trustworthiness of each source. In round two, questions regarding social and economic effects on households were added, for example, loss of employment, skipping meals, household costs, and gender-based violence or tensions experienced in the household. In round three, more detailed questions were added; such as how often participants are skipping meals. Each surveyor completed 10-15 surveys per day, and all phone numbers were tried up to three times if not reached on the first attempt. Questions were framed to reduce bias as much as possible. Data were collected using Open Data Kit (ODK) and exported to Stata v15 for analysis.

Ethical Approval

We received expedited ethical approval via amendments to existing protocols to re-contact respondents for the first two rapid surveys given the urgent need for information during this pandemic. The ongoing COVID-19 KAP survey cohort was approved by the Population Council IRB (p936) and AMREF ESRC (P803/2020). A research permit was issued by the National Commission for Science, Technology and Innovation (NACOSTI) in Kenya (P/20/5010). For AGI-K and NISITU, participants gave written informed consent, for the COVID-19 survey we verbal consent was given over the phone prior to administering the survey.

Participant Involvement

It was not possible to involve participants in the study design or interpretation of results, due to the rapid response required around COVID-19, the inability to engage face to face or hold events, and that no funds were allocated for this.

For peer review only - http://bmjopen.bmj.com/site/about/guidelines.xhtml

Questionnaires and reports are publicly available, with the full de-identified dataset available upon request.

Data Analysis

Data from AGI-K and NISITU 2019 surveys were merged with rounds one, two, and three of the COVID-19 survey for additional information on households characteristics. The overall analysis here focuses on the three rounds of the COVID-19 survey; we incorporated basic information from the pre-COVID-19 surveys (such as wealth quintile, household location, household composition), but otherwise comparisons over time refer to the COVID-19 surveys since the same respondent was interviewed. Key economic, social, and health variables were tabulated by survey round and gender. The survey question asked participants to self identify their sex as male or female; throughout this paper we will refer to respondents as men and women to illustrate that we explore how the pandemic impacts gender (the socially constructed characteristics of men and women) not biological sex.

Key economic variables included reporting increased food prices, loss of income/employment and skipping a meal due to the pandemic. Key social variables included seeing family and friends less due to COVID-19, or taking on more chores such as cooking, cleaning, and childcare. Participants were asked if they experienced more arguing, tension, household violence, or fear their partner would harm them due to COVID-19 (four binary questions). Key health variables focused on whether participants reported that in the last two weeks, they had required a health service (e.g., for malaria, acute illness, immunization, or others) but did not seek healthcare for themselves or their children. For analysis, participants who said yes to one or more of these questions were considered at risk of household violence. Very few questions had missing responses.

Linear probability regression models were constructed to explore: 1) factors associated with skipping meals due to COVID-19, 2) factors associated with risk of household violence and 3) factors associated with forgoing any health services due to COVID-19. Bivariate models were constructed first followed by a fully adjusted model. Fully adjusted models control for gender, age, wealth quintile, and informal settlement where the participant resides. In the models for skipping meals and forgoing health services, data were available for both April and May; in these models, clustering by participant was specified to allow the errors for the same participant to be correlated across each round over time. For the household violence risk model, these data were only collected from one time point (May). Models were then run stratified by gender. Linear probability regression models produce similar results to logistic regression, but allow for easier interpretation of the results in percentage points (pp).

RESULTS

Characteristics of respondents

A total of 2,009 individuals were surveyed in March, 1,761 in April (88% of round one), and 1,745 in May (99% of round two participants; 87% of round one participants). Overall, participants were 63% women, with an average age of 36.3 (SD=±11·4) (Table 1).

Table 1: Demographic characteristics of adult study sample selected in March 2020

Demographics	Women N (%)	Men N (%)	Total
**N**	1258 (62·8%)	747 (37·2%)	2009 (100%)
**Age**			
**18 – 24 years**	252 (20·0%)	181 (24·3%)	433 (21·6%)
**25 – 34 years**	285 (22·7%)	109 (14·6%)	394 (19·6%)
**35 – 44 years**	457 (36·3%)	243 (32·6%)	700 (34·8%)
**45 years and over**	264 (21·0%)	457 (36·3%)	477 (23·7%)
**Slum**			
**Dandora**	242 (19·2%)	217 (29·1%)	459 (22·9%)

For peer review only - http://bmjopen.bmj.com/site/about/guidelines.xhtml

**Huruma**	208 (16·5%)	67 (9·0%)	275 (13·7%)
**Kariobangi**	223 (17·7%)	191 (25·6%)	414 (20·6%)
**Kibera**	322 (25·6%)	118 (15·8%)	440 (21·9%)
**Mathare**	263 (20·9%)	153 (20·5%)	416 (20·7%)
**Education**			
**No School**	59 (4·7%)	14 (1·9%)	73 (3·6%)
**Primary**	572 (45·5%)	221 (29·7%)	793 (39·6%)
**Secondary**	518 (41·2%)	360 (48·3%)	878 (43·8%)
**Higher Education**	107 (8·5%)	150 (20·1%)	257 (12·8%)
**Marital Status**			
**Married**	632 (50·4%)	538 (72·2%)	1170 (58·5%)
**Single**	313 (24·9%)	189 (25·4%)	502 (25·1%)
**Divorced/Separated Widowed**	310 (24·7%)	18 (2·4%)	328 (16·4%)
**Respondent is head of household**	519 (41·3%)	619 (83·0%)	1,138 (56·8%)
**Household size**			
**1-2 people**	85 (6·8%)	186 (24·9%)	271 (13·5%)
**3-4 people**	436 (34·7%)	188 (25·2%)	624 (31·1%)
**5-6 people**	485 (38·6%)	254 (34·1%)	739 (36·9%)
**7+ people**	252 (20·0%)	118 (15·8%)	370 (18·5%)

Most participants had completed secondary school (44%) and most were married (59%), however there was variation by gender with men more likely to have achieved a higher educational level and more likely to be married. More than half of survey respondents reported they were the head of household, more so for men (83%) than women (41.3%). Between April and May 2020, significant changes in some social, health, and economic effects were noted. Almost half of participants reported complete loss of income (43% in April vs 36% in May), experiencing more violence in the household (5% in April vs 3% in May), and increased reporting of skipping meals in the last week due to COVID-19 (74% vs 68%) (Table 2). Despite some changes between round 2 and 3, most differences by gender stayed the same, for example, the proportion of women skipping meals was higher than men in both survey rounds.

Table 2: Economic, social, and health effects of mitigation measures in April (Round 2) and May (Round 3)

	**April (Round 2)**			**May (Round 3)**			**round p-value**
	**Women**	**Men**	**p value**	**Women**	**Men**	**p value**	
Economic effects							
Increase in food prices	870 (78·3%)	498 (76·6%)	0·41	927 (84·0%)	522 (81·3%)	0·14	<0·001
Complete loss of income	422 (38·0%)	216 (33·2%)	0·05	515 (46·7%)	230 (35·8%)	<0·001	<0·001
Skipped a meal this week due to COVID-19	775 (70·9%)	402 (62·9%)	0·001	838 (77·0%)	433 (68·0%)	<0·001	<0·001
Social effects							
See family less	607 (54·6%)	382 (58·8%)	0·09	621 (56·3%)	360 (56·1%)	0·93	0·946
See my friends less	968 (87·1%)	565 (86·9%)	0·9	1,018 (92·3%)	565 (88·0%)	0·003	0·001
Risk of household violence							
More tensions in the household	434 (39·1%)	211 (32·5%)	0·01	400 (36·3%)	199 (31·0%)	0·03	0·166
More violence experienced inside the house	38 (3·5%)	20 (3·1%)	0·7	68 (6·2%)	18 (2·8%)	0·002	0·019
More time arguing in the household	-	-	-	307 (27·8%)	141 (22·0%)	0·007	NA
More fear partner will harm you	-	-	-	74 (6·7%)	29 (4·5%)	0·06	NA

For peer review only - http://bmjopen.bmj.com/site/about/guidelines.xhtml

Combined into risk of household violence	-	-	-	495 (44·9%)	248 (38·6%)	0·01	NA
Household chores							
More housework (general)	747 (67·2%)	330 (50·1%)	<0·001	-	-	-	NA
More time spent cooking	-	-	-	539 (48·9%)	152 (23·7%)	<0·001	NA
More time spent cleaning	-	-	-	670 (60·7%)	162 (25·2%)	<0·001	NA
More time spent taking care of children	-	-	-	727 (65·9%)	230 (35·8%)	<0·001	NA
Neighborhood crime, violence							
Increased crime in the neighborhood	426 (38·3%)	222 (34·2%)	0·08	557 (50·5%)	294 (45·8%)	0·06	<0·001
More violence experienced outside the house	168 (15·1%)	92 (14·2%)	0·58	263 (23·8%)	146 (22·7%)	0·6	<0·001
Health effects							
Not purchasing sanitary pads (women only)	405 (36·5%)	-	NA	451 (41·0%)	-	NA	0·030
Not accessing needed health care/medicines	124 (11·2%)	30 (4·6%)	<0·001	117 (10·6%)	38 (5·9%)	0·001	1·000

There was no change in the proportion reporting that they were not seeking health services they needed, this was 9% in both April and May, but overall higher for women compared to men. In round three, questions also assessed measures of stigma related to COVID-19 infection, with significantly more women than men reporting that if infected people would gossip about them, and treat their family badly. Women were also less likely to say people would bring them food or medicine if needed (Figure 1).

Food Security

Overall the majority of participants reported skipping a meal in the last week due to COVID-19, with some variation in the characteristics of those who skipped a meal. In fully adjusted models for April and May survey rounds, women were 6 pp more likely than men to report skipping meals in the past week due to COVID-19 (Coeff: 0·055; 95% confidence interval 0·016, 0·094) (Table 3).

Table 3: Linear regression models of factors associated with skipping meals in the last week due to COVID-19

**VARIABLES**	**Unadjusted models Coefficients (95% CI)**	**Adjusted Model[1] Coefficients (95% CI)**	**Adjusted Model: Men Only Coefficients (95% CI)**	**Adjusted Model: Women only Coefficients (95% CI)**
Observations		3,451	1,274	2,177
Women (vs men)	0·085 (0·049, 0·122)***	0·055 (0·016, 0·094)***	··	··
Age category				
18-24 years	REF	REF	REF	REF
25-34 years	0·147 (0·092, 0·202)***	0·050 (-0·013, 0·114)	-0.051 (-0.160, 0.058)	0.120 (0.040, 0.199)***
35-44 years	0·150 (0·100, 0·199)***	0·047 (-0·018, 0·112)	-0.080 (-0.201, 0.041)	0.110 (0.030, 0.190)***
45+ years	0·087 (0·032, 0·141)***	-0·002 (-0·071, 0·067)	-0.107 (-0.232, 0.018)*	0.049 (-0.037, 0.135)
Marital status				
Married	REF	REF	REF	REF
Single	-0·109 (-0·153, -0·065)***	-0·059 (-0·118, -0·000)**	-0.178 (-0.289, -0.066)***	-0.008 (-0.076, 0.061)
Divorced/ separated/ widowed	0·081 (0·039, 0·123)***	0·077 (0·031, 0·122)***	0.081 (-0.101, 0.264)	0.080 (0.032, 0.128)***
Household Size				
1-2 people	REF	REF	REF	REF
3-4 people	0·048 (-0·012, 0·108)	-0·008 (-0·069, 0·053)	0.003 (-0.086, 0.091)	-0.043 (-0.133, 0.047)
5-6 people	0·111 (0·054, 0·168)***	0·049 (-0·011, 0·110)	0.070 (-0.018, 0.159)	0.018 (-0.072, 0.107)

For peer review only - http://bmjopen.bmj.com/site/about/guidelines.xhtml

7+ people	0·112 (0·048, 0·176)***	0·065 (-0·001, 0·132)*	0.104 (0.005, 0.203)**	0.032 (-0.064, 0.128)
Complete loss of income due to COVID-19	0·172 (0·141, 0·203)***	0·154 (0·125, 0·184)***	0.200 (0.149, 0.251)***	0.125 (0.089, 0.162)***
Survey Round (2 vs 3)	0·057 (0·027, 0·088)***	0·049 (0·023, 0·074)***	0.048 (0.004, 0.092)**	0.051 (0.020, 0.083)***

[1]Adjusted models also control for informal settlement where participant resides
*p<0.05; ** p<0.01; *** p<0.001

Those who reported a complete loss of income were 15 pp more likely to skip a meal (Coeff: 0·154; 95% CI 0·125, 0·184). Compared with married couples, participants who were single were less likely to report skipping meals (Coeff: -0·059; 95% CI -0·118, -0·000) and those who were divorced or widowed more likely to report skipping meals (Coeff: 0·077; 95% CI 0·031, 0·122). Reportedly skipping meals due to COVID-19 also increased from April to May survey rounds; compared with April, participants in May were 5 pp more likely to report skipping a meal (Coeff: 0·049; 95% CI 0·023, 0·074).
In models stratified by gender, both men and women were more likely to skip meals if they lost employment. Among women respondents, women who are divorced, widowed, or separated were more likely to skip a meal than women who are married (Coeff: 0·080; 95% CI 0·032, 0·128). Among men, having a larger household with more members was associated with an increased probability of skipping meals.

Risk of household violence

In May, households reported more tension, arguing, violence, or fear their partner would harm them; combined these indicate increased risk of household violence. In fully adjusted models, women were 8 pp more likely to report concerns regarding household tension and violence (Coeff: 0·079; 95% CI 0·028, 0·130) (Table 4).

Table 4: Linear regression models of factors associated with reporting increased risk of household violence

VARIABLES	Unadjusted Models Coefficients (95% CI)	Adjusted Model[1] Coefficients (95% CI)	Men Coefficients (95% CI)	Women Coefficients (95% CI)
Observations		1,775	636	1,085
Women (vs men)	0·062 (0·015, 0·110)**	0·079 (0·028, 0·130)***	..	..
Age				
18-24 years	REF	REF	REF	REF
25-34 years	0·121(0·048, 0·193)***	0·034 (-0·055, 0·122)	-0.152 (-0.296, -0.008)**	0.131 (0.016, 0.245)**
35-44 years	0·081 (0·017, 0·144)**	0·015 (-0·073, 0·104)	-0.038 (-0.191, 0.114)	0.044 (-0.069, 0.156)
45+ years	0·015 (-0·054, 0·084)	-0·019 (-0·112, 0·074)	-0.106 (-0.265, 0.052)	0.026 (-0.094, 0.147)
Marital status				
Married	REF	REF	REF	REF
Single	-0·089 (-0·145, -0·034)***	-0·073 (-0·152 - 0·006)*	-0.194 (-0.336, -0.053)***	0.011 (-0.088, 0.109)
Divorced/ separated/ widowed	-0·090 (-0·152, -0·026)***	-0·106 (-0·172, -0·039)***	-0.073 (-0.314, 0.168)	-0.078 (-0.151, -0.005)**
Has skipped a meal this week due to	0·198 (0·147, 0·250)***	0·164 (0·111, 0·218)***	0.172 (0.090, 0.254)***	0.140 (0.069, 0.212)***

For peer review only - http://bmjopen.bmj.com/site/about/guidelines.xhtml

For peer review only

COVID-19				
Complete loss of income	0·102 (0·055, 0·148)***	0·066 (0·019, 0·113)***	0.140 (0.063, 0.218)***	0.035 (-0.025, 0.095)

[1]Adjusted models also control for informal settlement where participant resides
*p<0.05; ** p<0.01; *** p<0.001

Married and/or cohabiting couples were most likely to report this concern. Participants who had skipped a meal in the last seven days due to COVID-19 were 16 pp more likely to report increased risk of household violence (Coeff: 0·164; 95% CI 0·111, 0·218) and those who reported lost income were 7 pp more likely to report household tension and violence (Coeff: 0·066; 95% CI 0·019, 0·114). In models stratified by gender, men who had completely lost their income due to COVID-19 were 14 pp more likely to report that they experienced increased tension in the household (Coeff: 0·140; 95% CI 0·063, 0·218) while this was not significant among women.

Forgoing needed health services

In fully adjusted models, women were 6 pp more likely to report not seeking health services they required (e.g., for acute illness, for malaria treatment, family planning) in the last 2 weeks (Coeff: 0·057; 95% CI 0·037, 0·076) (Table 5).

Table 5: Linear regression models of factors associated with forgoing health services in the last two weeks

VARIABLES	Unadjusted Models	Adjusted Model[1]
Observations		3,455
Women (vs men)	0·056 (0·037, 0·076)***	0·057 (0·037, 0·076)***
Age (18-24 years REF)		
18-24 years	REF	REF
25-34 years	0·011 (-0·018, 0·041)	-0·008 (-0·042, 0·026)
35-44 years	-0·013 (-0·039, 0·103)	-0·025 (-0·053, 0·003)*
45+ years	-0·001 (-0·029, 0·027)	-0·003 (-0·034, 0·028)
Skipped a meal this week due to COVID-19	0·060 (0·040, 0·081)***	0·049 (0·030, 0·068)***
Complete loss of income	0·051 (0·032, 0·071)***	0·042 (0·020, 0·064)***
Survey Round (2 vs 3)	0·001 (-0·017, 0·020)	-0·004 (-0·022, 0·014)

[1]Adjusted models also control for informal settlement where participant resides
*p<0.05; ** p<0.01; *** p<0.001

Those who had skipped a meal due to COVID-19 were 5 pp more likely to have forgone health services (coeff: 0·049; 95% CI 0·030, 0·068) and those who had completely lost employment were also more likely to skip necessary health services (coeff: 0·042; 95% CI 0·020, 0·064). There was no significant difference between April (round 2) and May (round 3) responses. Among those that skipped health services, over half said the reason was cost (52%). Participants who skipped health services were most likely to say the service they skipped was for any acute illness (27% in April, 21% in May), followed by seeking refills for any medications taken (19% in April, 20% in May).

For peer review only - http://bmjopen.bmj.com/site/about/guidelines.xhtml

Receiving Assistance

Between April and May survey rounds, the proportion of respondents receiving any assistance in the previous week tripled (7% in April to 21% in May); however, the proportion who had received assistance reporting that the assistance received covered their current needs decreased (47% in April, to 38% in May) (Figure 2). Most participants reported receiving soap or hand sanitizer, followed by food. When asked their single biggest need not being met, the main two items reported were food (94% in April, 86% in May) and cash (45% in April, 48% in May). Items were received from the government, NGO's, good Samaritans/corporate sponsorship, or religious institutions.

DISCUSSION

Overall, women in informal settlements in Nairobi are disproportionately affected by mitigation policies implemented to reduce the spread of COVID-19. Compared to men, women are more likely to report they have skipped meals, report heightened risk of household violence, and to report forgoing necessary healthcare services (for themselves or their children) due to the pandemic. Women are also more likely to report that if infected with COVID-19, they would experience stigma and have less social support. Between April and May, the proportion of respondents reporting that they are receiving government or NGO assistance increased, but the proportion reporting that this assistance is enough to meet their basic needs decreased. Our findings suggest that three months into the pandemic response, households in urban informal settlements in Nairobi are already experiencing significant economic, social, and health related harms. Women are disproportionately being impacted, and the effects of lost income, increased household chores, and social isolation may compound to exacerbate and reinforce existing gender inequalities. Without intervention, the effects on health and well-being may be severe and set back progress made on gender inequality to date. As Kenya begins a phased reopening (October 2020), it will be critical to support women to return to the workplace, to school, and to facilitate improved gender dynamics as well as access to support services for GBV survivors.

Economic Impacts

Our study identified the serious threat of food insecurity three months into the pandemic response in Nairobi, with almost three quarters of all study participants reporting they skipped a meal in the previous week due to COVID-19, higher for women than men. In crisis settings, women disproportionately bear the brunt of the effects of food insecurity with both short- and long-term implications for health and well-being.[12,33,34] Due to pandemic mitigation policies and movement restrictions, county government entities across Kenya have begun to close local, informal, outdoor markets where a majority of food is distributed.[35] These disruptions are likely to be causing supply shortages and price increases including in urban areas and reportedly making it difficult to access food, especially for families who cannot afford the higher food prices due to lost unemployment.[12,35] An African Union report projects that up to 20 million jobs could be lost in the region due to COVID-19.[36] Our study found that divorced women are at a higher risk of food insecurity compared to married and single women. This is perhaps due to the challenges a divorced woman faces as the single earner in the household while still also responsible for household chores including taking care of children, cleaning, laundry, and cooking. [37,38] Further research should explore this connection.

Social Impacts

Our study found that overall households are experiencing increased risk of household violence. This may be related to forced time spent in the home, widespread and sudden loss of employment leading to increased tension, and limited access to GBV support programs. During COVID-19 lockdowns in China, domestic violence reports tripled as people were forced to stay home.[23,39] During the Ebola outbreak in West Africa, GBV support initiatives were defunded due to prioritization of infection control, while in South Africa, rumors about COVID-19 led to a drop in utilization of GBV support programs.[39,40] Multiple studies have found that intimate partner violence risk increases when employment is lost,

For peer review only - http://bmjopen.bmj.com/site/about/guidelines.xhtml

including when women earn more than their partners or during socioeconomic hardship due to conflict.[41–43] This supports our finding that men surveyed reported more household tension, arguing and household violence risk if they had lost employment due to COVID-19. Our findings also indicate that increased food insecurity might be associated with increased risk of violence; studies in South Africa and Ethiopia prior to COVID-19 that found a significant link between household food insecurity status and increased risk of violence support this connection.[44,45] To date, the Kenyan government has not coordinated any national response, such as food distributions, cash transfers, or GBV support. The pandemic may exacerbate existing gender inequalities and roles, risking the fragile progress made to date on these issues. As Kenya begins a phased reopening it is critical to understand how the pandemic is shaping gender dynamics and how to address disparities moving forward.

Health Impacts

In addition to food insecurity and household violence, there is concern that COVID-19 will lead to adverse secondary health effects if routine care including immunization, nutrition, sexual and reproductive health, and antenatal care are not utilized. More women than men reported that they had forgone health services due to the pandemic, potentially reflecting that women are responsible for seeking care for themselves and for their children. The main reason respondents gave for skipping a necessary health service was that they could not afford the service. People may not be able to afford necessary care due to unemployment and rising costs of food and other basic necessities. In 2013, the Kenyan Ministry of Health reported that 83 percent of Kenyans were not financially able to cover healthcare costs and 1.5 million Kenyans are driven into poverty each year because of this.[46] The economic insecurity that will result from COVID-19 will likely reverse any progress on this issue, causing potential outbreaks of other diseases and worsening health outcomes. Respondents also cited to a lesser degree concerns regarding stigma if infected and access challenges. Participants in the survey report clinics are reducing patient volume to reduce risk of infection. On the demand side, people may avoid seeking care if they are concerned they will get the disease, or that they will be forced to quarantine. According to the World Health Organization, people who fear stigma related to COVID-19 infection may be driven to hide their illness, delay seeking healthcare, and be less likely to adopt healthy behaviors.[47] Women in our study report forgoing critical health services for themselves and for their children, signaling the potential for secondary adverse health effects. The government and other stakeholders must prioritize ensuring affordable and accessible health services in a healthcare setting that is safe for providers and patients.

Study Limitations

This study has some limitations. First, we sampled from participants from AGI-K and NISITU cohorts, meaning that the COVID-19 cohort sample is not representative of all households but rather households in which an adolescent resides. This is because the initial cohorts (AGI-K and NISITU) were designed for different purposes, but allowed us a rich dataset including mobile phone numbers to sample from. Therefore, for example, a household with only an elderly person or only early primary school aged children, would not have been eligible for inclusion in the COVID-19 survey. Second, all of the questions are self-reported, so there may be some bias in how participants respond to questions or recall bias. Relatedly, we interviewed one adult per household (whoever answered the phone), so their responses may not reflect all views or experiences of every household member. Other challenges relate to the phone-based nature of this survey. Since this was a phone-based survey, we faced some challenges verifying the respondent and had to rely on their responses. There also may have been some bias from who we could reach by phone, as some phone numbers never picked up. Another potential challenge with phone-based data collection is privacy; respondents may not have answered each question honestly if they were at home with others nearby. We tried to frame sensitive questions as yes/no responses so that respondents could answer honestly without others hearing the topic.

Recommendations

To combat the negative economic, social, and health related impacts of COVID-19 mitigation measures, initiating government assistance in the form of cash transfers or food distributions may be critical, along with increasing safe and affordable access to needed health services to prevent secondary outbreaks. To date, there has not been a coordinated national response to the pandemic; any interventions are run by NGO's (primarily working with their constituents prior to COVID-19), by religious institutions, and some regional leadership. Potential interventions to consider may include direct cash or asset transfers specifically to women, potentially utilizing community-based women's groups as existing infrastructure for social and economic support so that the support is local and accessible. Direct cash transfer initiatives in other African countries show that efforts to target assistance for social protection and GBV directly to women and girls are more effective and worth exploring in this scenario.[48,49] Community-based women's groups have been shown to play an integral and effective role in addressing local health needs, including one study that found they reduced the burden of HIV in Zimbabwe; with proper precautions these groups may be applied effectively in COVID-19 initiatives.[50–52] It is crucial to ensure that community health workers, mainly women themselves, are protected on the job and also able to provide gender sensitive services, including antenatal care and family planning. Organizations such as UNFPA are fighting for these critical sexual and reproductive health services to be considered 'essential' so that resources and staffing are not reallocated due to COVID-19;[53–55] disruptions in these services have potentially devastating consequences for women and their families. Lastly, GBV hotlines or digital technologies (such as mobile-phone based apps) for violence prevention may increase accessibility to critical social services, but more research is required on their effectiveness and ability to reach the most vulnerable women.[56] Initiating these types of measures will also help people sustain COVID-19 prevention measures over the longer term, for example, enabling them to safely stay home if another lockdown is implemented. These programs can also ensure that women and their families are able to resume their pre-COVID-19 trajectory and that progress toward development goals to date are not lost or reversed. Attention to the needs of women in these settings is critical as they are disproportionately at risk. Additional research is necessary to understand how the pandemic may shape gender dynamics and power structures in the short- and long-term, identifying opportunity for intervention and policy to continue to reduce inequalities. Our findings are likely to be broadly applicable to many other urban African contexts where there are large urban slums and strict mitigation policies.

CONCLUSION

Our findings suggest that three months into the COVID-19 pandemic and mitigation response, households in informal settlements are facing severe adverse social and economic effects, with women disproportionately impacted. Governments around the world are taking steps to balance the risks of COVID-19 transmission against the severe economic and social toll that will result from prolonged economic disruption. In the short-term, our findings highlight the urgent need to address rising unemployment, food insecurity, risk of GBV, all of which are more likely to be reported in this survey by women. Despite ongoing transmission of COVID-19, Kenya is entering a phased reopening. There is potential for additional disruptions if lockdowns or other mitigation policies return. Therefore, it will be critical to offer support programs targeted to the most at risk households and individuals as short-term stop gap measures, and in the longer term will be important to develop policies and programs to support women as the pandemic has threatened gender inequity gains made to date . Future research and policy should address the long-term effects of the pandemic, to understand how this event affected the life trajectories of urban women in informal slums. Any larger coordinated policies or interventions must be gender-sensitive to address the specific needs and situations of women.

For peer review only - http://bmjopen.bmj.com/site/about/guidelines.xhtml

Author contributions: JP helped develop the study design, instrument, conducted data analysis and led drafting of the manuscript. KA led the study design, implementation, supported with developing the analysis plan, and provided input on the manuscript. NR conducted the literature review, supported with data analysis, and supported drafting of the manuscript and tables. TA, BK, and RO supported study design, implementation, and provided input on the manuscript. JBT supported with study design, data management and analysis, and input on the manuscript. DM, EM, FM, and MN supported with study design, data collection, field team supervision, and provided input the manuscript. TDN conceived of the study, helped develop the study design, analysis plan, and provided input on the manuscript.

Competing Interests: None to declare.

Funding: UK Department for International Development through Innovations for Poverty Action Peace & Recovery COVID-19 rapid response grant. (Grant #MIT0019-X15)

Data Sharing Statement: data are available upon request.

Acknowledgements: the authors would like to acknowledge all of the work done by our field team to collect these surveys.

For peer review only

REFERENCES

1 Cucinotta D, Vanelli M. WHO declares COVID-19 a pandemic. Acta Biomed. 2020. doi:10.23750/abm.v91i1.9397
2 World Health Organization. New WHO estimates: Up to 190 000 people could die of COVID-19 in Africa if not controlled. WHO Africa. 2020.https://www.afro.who.int/news/new-who-estimates-190-000-people-could-die-covid-19-africa-if-not-controlled
3 Harrisburg. K. Half urban population of sub-Saharan Africa still living in slums: study. Reuters. https://www.reuters.com/article/us-africa-housing-disease/half-urban-population-of-sub-saharan-africa-still-living-in-slums-study-idUSKCN1R823K
4 Snyder RE, Marlow MA, Riley LW. Ebola in urban slums: The elephant in the room. Lancet Glob. Heal. 2014. doi:10.1016/S2214-109X(14)70339-0
5 Quaife M, Zandvoort K van, Gimma A, *et al.* The impact of COVID-19 control measures on social contacts and transmission in Kenyan informal settlements. *medRxiv* Published Online First: 2020. doi:10.1101/2020.06.06.20122689
6 Mehtar S, Preiser W, Lakhe NA, *et al.* Limiting the spread of COVID-19 in Africa: one size mitigation strategies do not fit all countries. Lancet Glob. Heal. 2020. doi:10.1016/S2214-109X(20)30212-6
7 Kabale N. Coronavirus: Kenya takes bold steps after cases rise to three. Dly. Nation. https://www.nation.co.ke/dailynation/news/coronavirus-kenya-takes-bold-steps-after-cases-rise-to-three-278692
8 Center. JHC-19 R. COVID-19 Case Tracker 2020. Available from https//coronavirus.jhu.edu/.
9 UN Habitat. Kenya Habitat Country Programme Document. (2018-2021). Nairobi, Kenya.: 2019. https://unhabitat.org/sites/default/files/2019/09/hcpd_kenya_2018_-_2021_0.pdf
10 Austrian K, Pinchoff J, Tidwell JB, *et al.* COVID-19 Related Knowledge, Attitudes, Practices and Needs of Households in Informal Settlements in Nairobi, Kenya. *SSRN Electron J* Published Online First: 2020. doi:10.2139/ssrn.3576785
11 Roberton T, Carter ED, Chou VB, *et al.* Early estimates of the indirect effects of the COVID-19 pandemic on maternal and child mortality in low-income and middle-income countries: a modelling study. *Lancet Glob Heal* Published Online First: 2020. doi:10.1016/s2214-109x(20)30229-1
12 The Lancet Global Health. Food insecurity will be the sting in the tail of COVID-19. *Lancet Glob Heal*;Volume 8.
13 Ruel MT, Garrett JL, Hawkes C, *et al.* The Food, Fuel, and Financial Crises Affect the Urban and Rural Poor Disproportionately: A Review of the Evidence. *J Nutr* Published Online First: 2010. doi:10.3945/jn.109.110791
14 World Health Organization. 2019 Novel Coronavirus Strategic Preparedness and Response Plan. 2020. https://www.who.int/publications/i/item/strategic-preparedness-and-response-plan-for-the-new-coronavirus
15 Corburn J, Vlahov D, Mberu B, *et al.* Slum Health: Arresting COVID-19 and Improving Well-Being in Urban Informal Settlements. *J Urban Heal* Published Online First: 2020. doi:10.1007/s11524-020-00438-6
16 Azcona G, Bhatt A, Ndugwa R. COVID-19 exposes the harsh realities of gender inequality in slums. https://data.unwomen.org/features/covid-19-exposes-harsh-realities-gender-inequality-slums
17 Wenham C, Smith J, Morgan R. COVID-19: the gendered impacts of the outbreak. Lancet. 2020. doi:10.1016/S0140-6736(20)30526-2
18 Emina J, Beguy D, Zulu EM, *et al.* Monitoring of health and demographic outcomes in poor urban settlements: Evidence from the Nairobi Urban Health and Demographic Surveillance System. *J Urban Heal* Published Online First: 2011. doi:10.1007/s11524-011-9594-1
19 Lafrenière J, Sweetman C, Thylin T. Introduction: gender, humanitarian action and crisis response. *Gend Dev*;:Pages 187-201.

For peer review only - http://bmjopen.bmj.com/site/about/guidelines.xhtml

20 Lucas B. Impacts of Covid-19 on inclusive economic growth in middle-income countries. *KD4 Help Rep 811 Bright UK Inst Dev Stud*
21 UN Women. Policy Brief: The Impact of COVID-19 on Women. 2020.
22 van Gelder N, Peterman A, Potts A, *et al.* COVID-19: Reducing the risk of infection might increase the risk of intimate partner violence. EClinicalMedicine. 2020. doi:10.1016/j.eclinm.2020.100348
23 World Health Organization. COVID-19 and violence against women: What the health sector / system can do. 2020. https://apps.who.int/iris/handle/10665/331699
24 Joska JA, Andersen L, Rabie S, *et al.* COVID-19: Increased Risk to the Mental Health and Safety of Women Living with HIV in South Africa. AIDS Behav. 2020. doi:10.1007/s10461-020-02897-z
25 No dog walking, alcohol sale during lockdown. South African Gov. News Agency.
26 Boulle A, Davies M-A, Hussey H, *et al.* Risk factors for COVID-19 death in a population cohort study from the Western Cape Province, South Africa. *Clin Infect Dis* Published Online First: 2020. doi:10.1093/cid/ciaa1198
27 Palaiodimos L, Kokkinidis DG, Li W, *et al.* Severe obesity, increasing age and male sex are independently associated with worse in-hospital outcomes, and higher in-hospital mortality, in a cohort of patients with COVID-19 in the Bronx, New York. *Metabolism* Published Online First: 2020. doi:10.1016/j.metabol.2020.154262
28 Jin JM, Bai P, He W, *et al.* Gender Differences in Patients With COVID-19: Focus on Severity and Mortality. *Front Public Heal* Published Online First: 2020. doi:10.3389/fpubh.2020.00152
29 Eggers R. WHO Kenya Coronavirus update. *World Heal Organ*
30 World Vision. Covid-19 Aftershocks: Secondary Impacts Threaten More Children'S Lives Than Disease Itself. 2020. https://www.wvi.org/publications/report/coronavirus-health-crisis/covid-19-aftershocks-secondary-impacts-threaten-more.
31 Colavita F, Biava M, Castilletti C, *et al.* Measles cases during ebola outbreak, West Africa, 2013–2106. Emerg. Infect. Dis. 2017. doi:10.3201/eid2306.161682
32 Austrian K, Muthengi E, Mumah J, *et al.* The Adolescent Girls Initiative-Kenya (AGI-K): Study protocol. *BMC Public Health* Published Online First: 2016. doi:10.1186/s12889-016-2888-1
33 Sasson A. Food security for Africa: An urgent global challenge. Agric. Food Secur. 2012. doi:10.1186/2048-7010-1-2
34 Modirwa S, Oladele OI. Food Security among Male and Female-headed Households in Eden District Municipality of the Western Cape, South Africa. *J Hum Ecol* Published Online First: 2012. doi:10.1080/09709274.2012.11906446
35 Kusmer A. Coronavirus — and locusts — threaten Kenya's food security. The World. https://www.pri.org/stories/2020-05-13/coronavirus-and-locusts-threaten-kenya-s-food-security
36 African Union. Impact of the Coronavirus (COVID-19) on the African Economy. 2020. https://icsb.org/wp-content/uploads/2020/04/Covid-19-impact-on-African-economy.pdf.pdf.pdf.pdf
37 Grant MJ, Pike I. Divorce, living arrangements, and material well-being during the transition to adulthood in rural Malawi. *Popul Stud (NY)* Published Online First: 2019. doi:10.1080/00324728.2018.1545919
38 Lawson DW, Schaffnit SB, Hassan A, *et al.* Father absence but not fosterage predicts food insecurity, relative poverty, and poor child health in northern Tanzania. *Am J Hum Biol* Published Online First: 2017. doi:10.1002/ajhb.22938
39 Gould C. Gender-Based Violence During Lockdown - Looking for Answers [analysis]. Mark. Watch. https://www.marketwatch.com/press-release/gender-based-violence-during-lockdown---looking-for-answers-analysis-2020-05-12
40 John N, Casey SE, Carino G, *et al.* Lessons Never Learned: Crisis and gender-based violence. *Dev World Bioeth* Published Online First: 2020. doi:10.1111/dewb.12261
41 Abramsky T, Lees S, Stöckl H, *et al.* Women's income and risk of intimate partner violence:

For peer review only - http://bmjopen.bmj.com/site/about/guidelines.xhtml

Secondary findings from the MAISHA cluster randomised trial in North-Western Tanzania. *BMC Public Health* Published Online First: 2019. doi:10.1186/s12889-019-7454-1
42 Rees S, Mohsin M, Tay AK, *et al.* Risk of perpetrating intimate partner violence amongst men exposed to torture in conflict-affected Timor-Leste. *Glob Ment Heal* Published Online First: 2018. doi:10.1017/gmh.2018.16
43 Cardoso LF, Gupta J, Shuman S, *et al.* What Factors Contribute to Intimate Partner Violence Against Women in Urban, Conflict-Affected Settings? Qualitative Findings from Abidjan, Côte d'Ivoire. *J Urban Heal* Published Online First: 2016. doi:10.1007/s11524-016-0029-x
44 Hatcher AM, Stöckl H, McBride RS, *et al.* Pathways From Food Insecurity to Intimate Partner Violence Perpetration Among Peri-Urban Men in South Africa. *Am J Prev Med* Published Online First: 2019. doi:10.1016/j.amepre.2018.12.013
45 Andarge E, Shiferaw Y. Disparities in intimate partner violence among currently married women from food secure and insecure urban households in South Ethiopia: A community based comparative cross-sectional study. *Biomed Res Int* Published Online First: 2018. doi:10.1155/2018/4738527
46 Ministry of Health. Nairobi: Government of Kenya. The Kenya household health expenditure and utilisation survey. 2013. https://www.healthpolicyproject.com/pubs/745_KHHUESReportJanuary.pdf
47 World Health Organization. Social Stigma associated with COVID-19. 2020. https://www.who.int/docs/default-source/coronaviruse/covid19-stigma-guide.pdf
48 Kilburn KN, Pettifor A, Edwards JK, *et al.* Conditional cash transfers and the reduction in partner violence for young women: An investigation of causal pathways using evidence from a randomized experiment in South Africa (HPTN 068): An. *J Int AIDS Soc* Published Online First: 2018. doi:10.1002/jia2.25043
49 Vincent K, Cull T. Impacts of social cash transfers: case study evidence from across southern Africa. *II Conferência IESE " Dinâmicas da Pobr e Padrões Acumulação Económica em Moçambique "* 2009.
50 Gregson S, Terceira N, Mushati P, *et al.* Community group participation:: Can it help young women to avoid HIV? An exploratory study of social capital and school education in rural Zimbabwe. *Soc Sci Med* 2004.
51 Kamau M, Mirie W, Kimani S, *et al.* Effect of community based health education on knowledge and attitude towards iron and folic acid supplementation among pregnant women in Kiambu County, Kenya: A quasi experimental study. *PLoS One* Published Online First: 2019. doi:10.1371/journal.pone.0224361
52 de Hoop T, Desai S, Siwach G, *et al.* Women's groups and CO oups and COVID-19: Challenges, engagement, and VID-19: Challenges, engagement, and opportunities. 2020.
53 UNFPA. COVID-19: A Gender Lens: Protecting Sexual and Reproductive Health and Rights, and promoting gender equality. 2020. https://www.unfpa.org/sites/default/files/resource-pdf/COVID-19_A_Gender_Lens_Guidance_Note.pdf
54 Ahmed SAKS, Ajisola M, Azeem K, *et al.* Impact of the societal response to COVID-19 on access to healthcare for non-COVID-19 health issues in slum communities of Bangladesh, Kenya, Nigeria and Pakistan: results of pre-COVID and COVID-19 lockdown stakeholder engagements. *BMJ Glob Heal* Published Online First: 2020. doi:10.1136/bmjgh-2020-003042
55 Beech P. The COVID-19 pandemic could have huge knock-on effects on women's health, says the UN. World Econ. Forum. 2020.https://www.weforum.org/agenda/2020/04/covid-19-coronavirus-pandemic-hit-women-harder-than-men/
56 Jewkes R, Dartnall E. More research is needed on digital technologies in violence against women. Lancet Public Heal. 2019. doi:10.1016/S2468-2667(19)30076-3

For peer review only - http://bmjopen.bmj.com/site/about/guidelines.xhtml

Figure Legends

Figure 1: Proportion of participants reporting stigma and social support related to COVID-19 by gender as measured in May (Round 3)

Figure 2: Proportion of participants reporting assistance received and specific types of support received, comparing April (Round 2) and May (Round 3)

For peer review only

For peer review only - http://bmjopen.bmj.com/site/about/guidelines.xhtml

Figure 1: Proportion of participants reporting stigma and social support related to COVID-19 by gender as measured in May (Round 3).

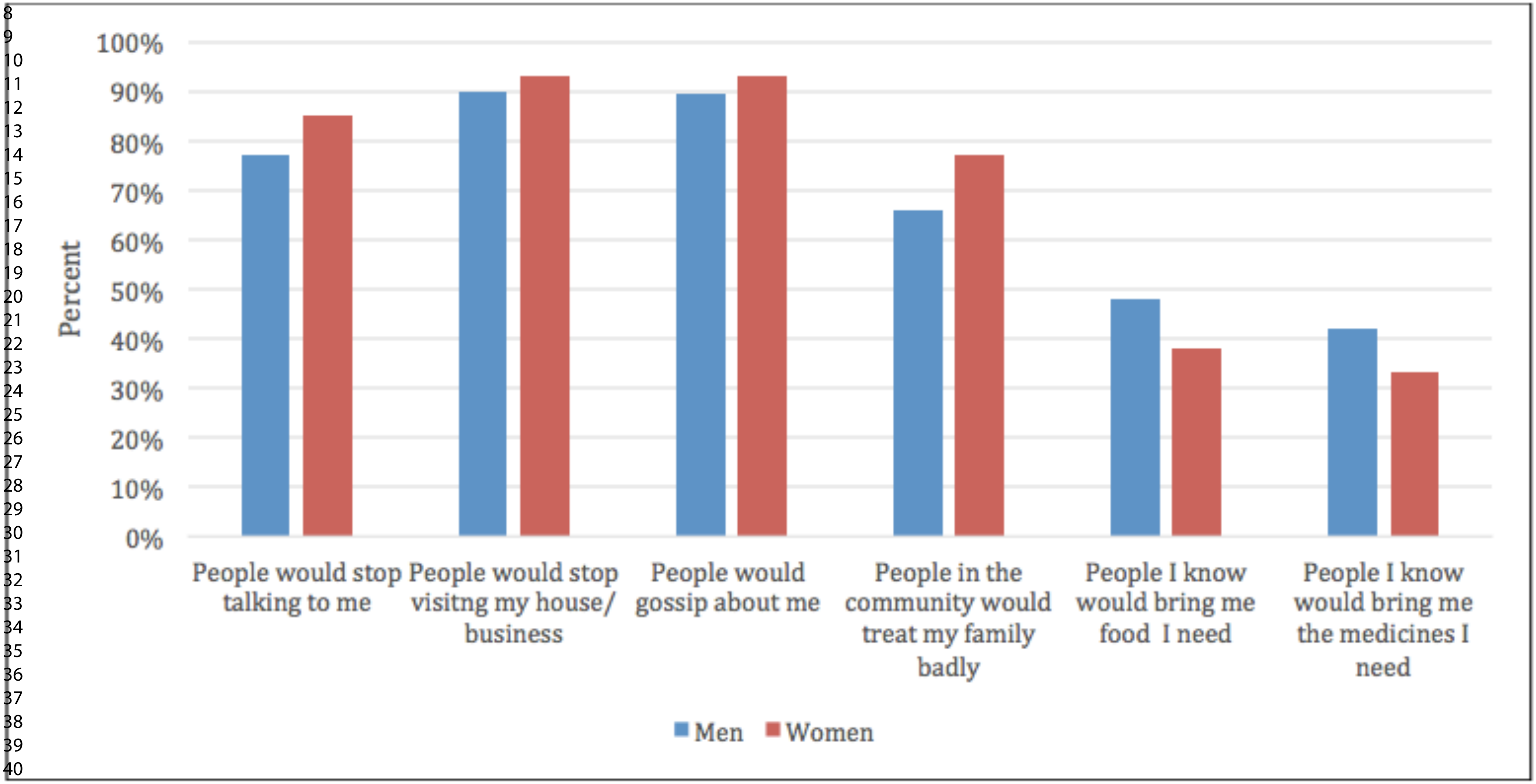

Figure 2: Proportion of participants reporting assistance received and specific types of support received, comparing April (Round 2) to May (Round 3)

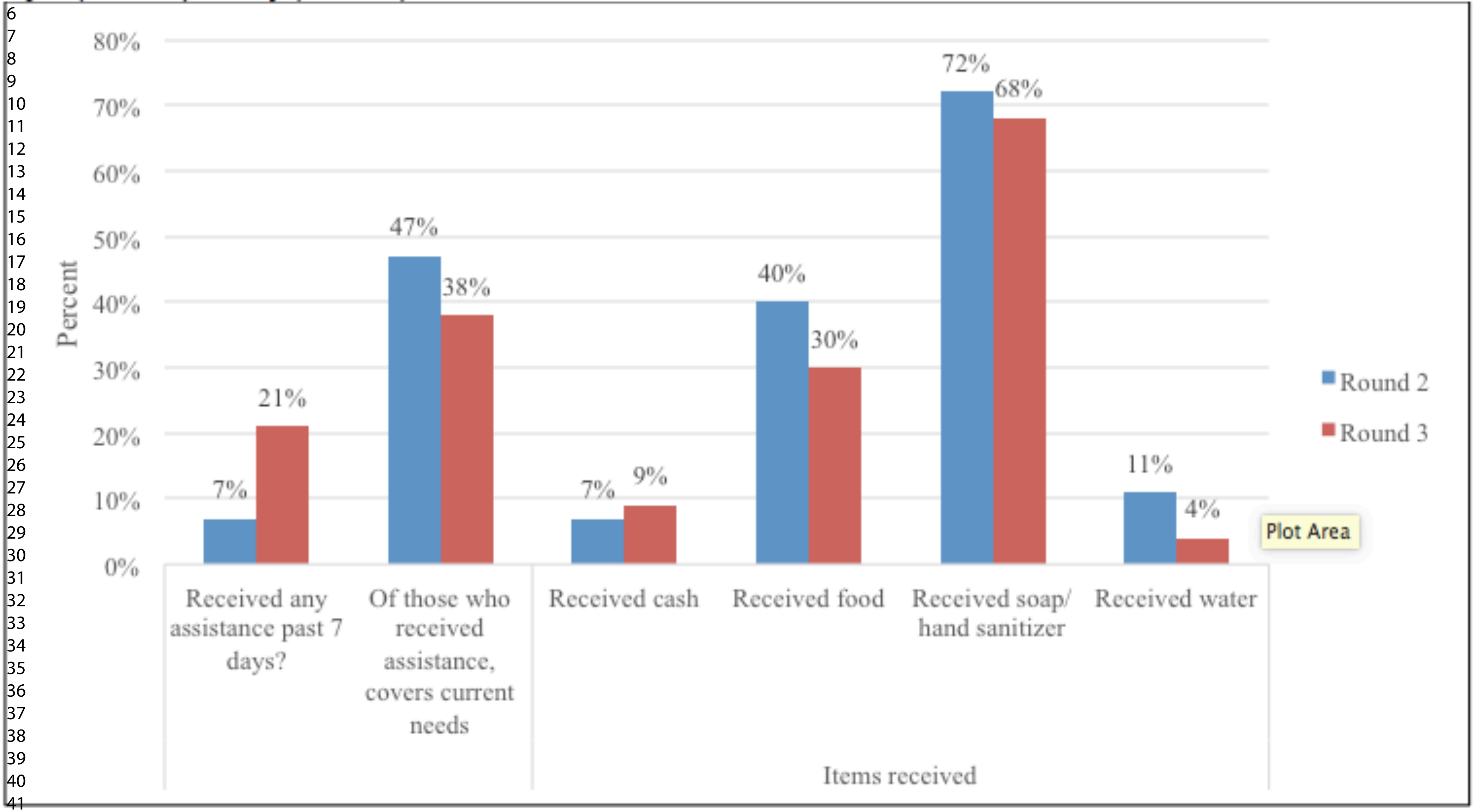

STROBE Statement—Checklist of items that should be included in reports of ***cohort studies***

	Item No	Recommendation	Page No
**Title and abstract**	1	(*a*) Indicate the study's design with a commonly used term in the title or the abstract	1
		(*b*) Provide in the abstract an informative and balanced summary of what was done and what was found	2
**Introduction**			
Background/rationale	2	Explain the scientific background and rationale for the investigation being reported	3-4
Objectives	3	State specific objectives, including any prespecified hypotheses	4
**Methods**			
Study design	4	Present key elements of study design early in the paper	4
Setting	5	Describe the setting, locations, and relevant dates, including periods of recruitment, exposure, follow-up, and data collection	4
Participants	6	(*a*) Give the eligibility criteria, and the sources and methods of selection of participants. Describe methods of follow-up	4-5
		(*b*) For matched studies, give matching criteria and number of exposed and unexposed	-
Variables	7	Clearly define all outcomes, exposures, predictors, potential confounders, and effect modifiers. Give diagnostic criteria, if applicable	5
Data sources/ measurement	8*	For each variable of interest, give sources of data and details of methods of assessment (measurement). Describe comparability of assessment methods if there is more than one group	5
Bias	9	Describe any efforts to address potential sources of bias	5
Study size	10	Explain how the study size was arrived at	4
Quantitative variables	11	Explain how quantitative variables were handled in the analyses. If applicable, describe which groupings were chosen and why	5
Statistical methods	12	(*a*) Describe all statistical methods, including those used to control for confounding	
		(*b*) Describe any methods used to examine subgroups and interactions	5
		(*c*) Explain how missing data were addressed	
		(*d*) If applicable, explain how loss to follow-up was addressed	
		(*e*) Describe any sensitivity analyses	
**Results**			
Participants	13*	(a) Report numbers of individuals at each stage of study—eg numbers potentially eligible, examined for eligibility, confirmed eligible, included in the study, completing follow-up, and analysed	6
		(b) Give reasons for non-participation at each stage	
		(c) Consider use of a flow diagram	
Descriptive data	14*	(a) Give characteristics of study participants (eg demographic, clinical, social) and information on exposures and potential confounders	6-7
		(b) Indicate number of participants with missing data for each variable of interest	
		(c) Summarise follow-up time (eg, average and total amount)	
Outcome data	15*	Report numbers of outcome events or summary measures over time	

For peer review only

Main results	16	(*a*) Give unadjusted estimates and, if applicable, confounder-adjusted estimates and their precision (eg, 95% confidence interval). Make clear which confounders were adjusted for and why they were included<br>(*b*) Report category boundaries when continuous variables were categorized<br>(*c*) If relevant, consider translating estimates of relative risk into absolute risk for a meaningful time period	7-10
Other analyses	17	Report other analyses done—eg analyses of subgroups and interactions, and sensitivity analyses	
**Discussion**			
Key results	18	Summarise key results with reference to study objectives	10-12
Limitations	19	Discuss limitations of the study, taking into account sources of potential bias or imprecision. Discuss both direction and magnitude of any potential bias	12
Interpretation	20	Give a cautious overall interpretation of results considering objectives, limitations, multiplicity of analyses, results from similar studies, and other relevant evidence	12
Generalisability	21	Discuss the generalisability (external validity) of the study results	12
**Other information**			
Funding	22	Give the source of funding and the role of the funders for the present study and, if applicable, for the original study on which the present article is based	12

*Give information separately for exposed and unexposed groups.

Note: An Explanation and Elaboration article discusses each checklist item and gives methodological background and published examples of transparent reporting. The STROBE checklist is best used in conjunction with this article (freely available on the Web sites of PLoS Medicine at http://www.plosmedicine.org/, Annals of Internal Medicine at http://www.annals.org/, and Epidemiology at http://www.epidem.com/). Information on the STROBE Initiative is available at http://www.strobe-statement.org.

For peer review only
